# Broadband infrared imaging governed by guided-mode resonance in dielectric metasurfaces

**DOI:** 10.1038/s41377-024-01535-w

**Published:** 2024-09-10

**Authors:** Ze Zheng, Daria Smirnova, Gabriel Sanderson, Ying Cuifeng, Demosthenes C. Koutsogeorgis, Lujun Huang, Zixi Liu, Rupert Oulton, Arman Yousefi, Andrey E. Miroshnichenko, Dragomir N. Neshev, Mary O’Neill, Mohsen Rahmani, Lei Xu

**Affiliations:** 1 Advanced Optics and Photonics Laboratory, Department of Engineering, School of Science & Technology, Nottingham Trent University, Nottingham, NG11 8NS UK; 2 ARC Centre of Excellence for Transformative Meta-Optical Systems (TMOS), Research School of Physics, The Australian National University, Canberra, ACT 2601 Australia; 3 School of Science & Technology, Nottingham Trent University, Nottingham, NG11 8NS UK; 4 School of Physics and Electronic Science, East China Normal University, Shanghai, 200241 China; 5 School of Physics, Nankai University, Tianjin, 300071 China; 6 Department of Physics, Imperial College London, London, SW7 2BW UK; 7 School of Engineering and Technology, University of New South Wales, Canberra, ACT 2600 Australia

**Keywords:** Imaging and sensing, Metamaterials, Nonlinear optics

## Abstract

Nonlinear metasurfaces have experienced rapid growth recently due to their potential in various applications, including infrared imaging and spectroscopy. However, due to the low conversion efficiencies of metasurfaces, several strategies have been adopted to enhance their performances, including employing resonances at signal or nonlinear emission wavelengths. This strategy results in a narrow operational band of the nonlinear metasurfaces, which has bottlenecked many applications, including nonlinear holography, image encoding, and nonlinear metalenses. Here, we overcome this issue by introducing a new nonlinear imaging platform utilizing a pump beam to enhance signal conversion through four-wave mixing (FWM), whereby the metasurface is resonant at the pump wavelength rather than the signal or nonlinear emissions. As a result, we demonstrate broadband nonlinear imaging for arbitrary objects using metasurfaces. A silicon disk-on-slab metasurface is introduced with an excitable guided-mode resonance at the pump wavelength. This enabled direct conversion of a broad IR image ranging from >1000 to 4000 nm into visible. Importantly, adopting FWM substantially reduces the dependence on high-power signal inputs or resonant features at the signal beam of nonlinear imaging by utilizing the quadratic relationship between the pump beam intensity and the signal conversion efficiency. Our results, therefore, unlock the potential for broadband infrared imaging capabilities with metasurfaces, making a promising advancement for next-generation all-optical infrared imaging techniques with chip-scale photonic devices.

## Abstract (web summary)

We experimentally demonstrate high-quality infrared imaging ranging from >1000 to 4000 nanometers with Si metasurfaces based on four-wave mixing (FWM) processes, by employing a pump beam at resonant position.

## Graphical abstract

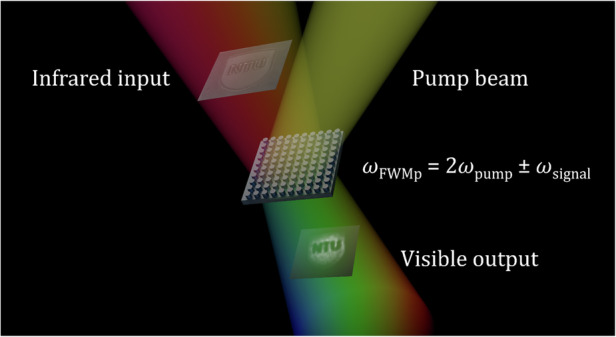

## Introduction

Nonlinear generation, a basic phenomenon of converting light from one frequency to a new frequency, has been extensively applied in various techniques and applications, including nonlinear imaging, quantum computing, and optical sensing^1^. Recently, metasurfaces, composed of subwavelength constituent resonant atoms, have been recognized as a novel and super-compact platform to achieve nonlinear generation at the nanoscale^2–4^. By exciting plasmonic resonances or multipolar resonances to obtain extremely strong light-matter interactions, nonlinear generation can be significantly enhanced within nonlinear metasurfaces^5–7^. Furthermore, the conversion efficiency of nonlinear metasurfaces is mainly determined by the field enhancement induced by resonances, unlike other nonlinear platforms, such as nonlinear crystals, fibers, and waveguides, in which the conversion efficiency is highly dependent on propagating loss and phase matching terms^8–10^. As a result, light in free space can directly couple into nonlinear metasurfaces and generate nonlinear signals, offering superiority for metasurfaces to be applied in the nonlinear imaging field.

Nonlinear imaging techniques, which utilize nonlinear generation to create and convert images, have enormous application potential in extensive fields, including sensing, night vision, and spectroscopy^11–14^. Nonlinear metasurfaces have the ability to manipulate the amplitude, phase, and directionality of nonlinear emissions in subwavelength pixels^15^. Due to this unique property, many works have reported encoding the amplitude and phase distribution for each subwavelength pixel of metasurfaces to achieve nonlinear holography^16–18^, imaging encoding^17,19^, nonlinear metalenses^20–22^, nonlinear light routing^23^. Nevertheless, the effect of phase change and amplitude manipulation mainly originates from resonances and thus highly restricts the operating wavelength range of nonlinear metasurfaces. Moreover, for applications like nonlinear holography and imaging encoding, typically, only one or a few predesigned images can be encoded within a single metasurface. Although some efforts have been devoted to employing tuneable metasurfaces in nonlinear imaging technique^24^, achieving tuneability and full control over each unit in the metasurface for displaying images remains an immense challenge.

To achieve nonlinear imaging of arbitrary objects, the concept of exploiting nonlinear metasurfaces for direct image conversion is currently emerging^11,12^. This approach removes the need to construct amplitude and phase distributions. Instead, metasurfaces for image conversion are designed to obtain high conversion efficiencies, reducing the dependence on high-power inputs. Several concepts, such as bound states in the continuum and nonlocal resonances, have been implemented to construct high-Q resonances and strong field enhancements, thereby boosting conversion efficiencies^25–32^. However, these strategies result in a narrow operating bandwidth for nonlinear metasurfaces, which highly limits the application potential of nonlinear imaging techniques.

In this paper, we utilize the four-wave mixing (FWM) process to bypass the limitation of the narrow operating bandwidth of high-Q resonances. By introducing a pump beam at high-Q resonant position, we successfully achieve the nonlinear imaging of arbitrary objects across the spectrum from near-infrared to mid-infrared based on the FWM process. Additionally, we calculate and measure the angle dependence of the transmission spectra for the Si metasurface, observing the topological resonance splitting of guided-mode resonances. The quadratic and linear power dependencies of two FWM processes on signal and pump beams are measured. The linear polarization dependence of the signal and pump beams on the FWM emission is also demonstrated experimentally and theoretically. Notably, we experimentally demonstrated that our FWM-based imaging platform allows for the detection of sample thickness based on the time delays of two pulsed lasers. The exploitation of FWM for nonlinear imaging based on metasurfaces opens new possibilities for developing compact, ultrathin metadevices for infrared imaging techniques.

## Results

**Fig. 1:**
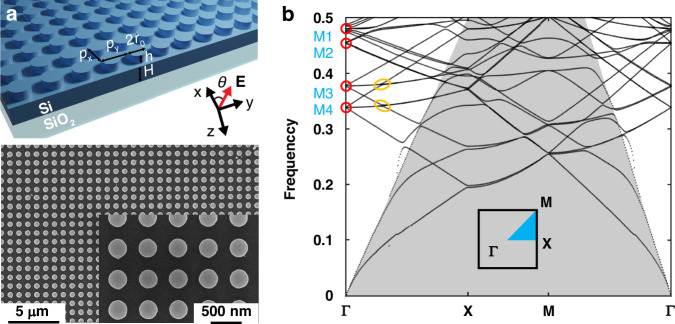
The fabricated Si metasurface and its band structure. **a** Top: The schematic diagram of Si metasurfaces. Bottom: SEM images of fabricated Si metasurface. **b** Calculated band structures for the Si metasurface with the positions of guided modes M1, M2, M3, and M4 (marked with red circles) and coupling regions (marked with orange circles)

In this work, we focus on the Si metasurface consisting of a Si film with Si nanodisks arranged periodically on top of the film, as depicted in Fig. 1a. The heights of the Si film and nanodisks are set to be *H* = 300 nm and *h* = 200 nm, respectively. The radius of the nanodisks is *r*_0_ = 150 nm. The lattice constants in the x and y directions *p*_*x*_ and *p*_*y*_ are both set to be 500 nm. In our simulations, the refractive indices of Si and SiO_2_ are set as 3.4 and 1.5, respectively. The *x*, *y*, *z* axes are set as shown in Fig. 1a with the polarization direction of the incident light defined as the angle *θ*, which is measured between the polarization direction and *x*-axis. By calculating the band structure of an infinite Si film and the Si metasurface under the discrete translational symmetry **F**(**r**) = **F**(**r** + *p*_*x*_**i** + *p*_*y*_**j**), the dispersion relation of guided modes can be obtained, as shown in Fig. 1b (for the metasurface) and Section I of Supplementary Material (for the film). Consequently, four guided modes M1, M2, M3, and M4 can be predicted with two coupling regions marked with red and orange circles obtained in the Si metasurface. It is important to note that the Si film itself intrinsically exhibits continuous translational symmetry instead of discrete translational symmetry. Here, we apply a virtual periodic boundary condition with a specified period to obtain the band structure. In general, the guided modes supported by the Si film can be designed at any spectral region by choosing the artificial period, providing a robust platform for the nanoscale light-matter interactions.

**Fig. 2:**
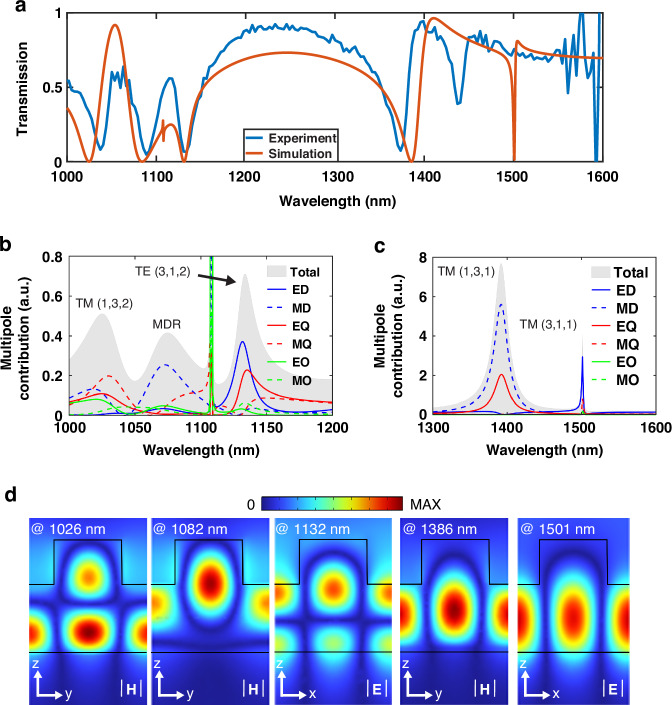
The properties of the Si metasurface, including the transmission spectra under normal incidence, its multipolar analysis, and resonant field distributions. **a** The measured and simulated transmission spectra of Si metasurfaces. **b** The calculated multipolar analysis of the Si metasurface from 1000 to 1200 nm. **c** The calculated multipolar analysis of the Si metasurface from 1300 to 1600 nm. **d** The calculated field distributions of TM(1,3,2), MD, TE(3,1,2), TM(1,3,1), and TE(3,1,1) under the *y*-polarized illumination

The continuous translational symmetry is transformed into discrete one by introducing the periodic array of Si nanodisks on top of the Si film to construct Si metasurfaces. The four guided modes M1-4 are successfully transformed into high-Q guided resonances under the external excitation of linearly-polarized light incidence, as shown in the measured and simulated transmission spectra (Fig. 2a). Here, based on the modified Fabry-Perot model, we define transverse electric-like modes and magnetic-like modes as TE(*l, m, n*) and TM(*l, m, n*), where *l*, *m*, *n* are the quantum numbers of the mode along the *x*, *y,* and *z* axes, respectively^33,34^. They correspond to the number of peaks of the electric/magnetic field within the domain along *x*, *y,* and *z* directions. In this way, the four guided-mode resonances can be defined as TE(3,1,1) at 1501 nm, TM(1,3,1) at 1386 nm, TE(3,1,2) at 1132 nm, and TM(1,3,2) at 1026 nm, with one additional Mie resonance which is a magnetic dipole resonance (MDR) introduced by Si nanodisks located at 1082 nm in the middle of TE(3,1,1) and TM(1,3,1). Furthermore, the band structure of a Si disk metasurface with the same thickness and the same unit cell size is also calculated in Section I of Supporting Information, which contains fewer eigenmodes (four modes) compared to the disk-film metasurface within the same range of spectrum. Importantly, owing to the perturbation of the silicon structure along the *z* direction, modes with different quantum numbers *n* are de-orthogonalized and enable the possibility of engineering the multiple content and radiation features of these modes through their couplings.

We further analyze the behaviors of these five resonances by performing the spherical multipolar analysis demonstrated in Fig. 2b, c and Section II of Supporting Information^35–37^. The ED, MD, EQ, MQ, EO, MO respectively represent the electric dipole (ED) and magnetic dipole (MD), electric quadrupole (EQ) and magnetic quadrupole (MQ), electric octupole (EO) and magnetic octupole (MO). Figure 2b indicates the Mie resonance is dominated by the magnetic dipole resonance, named as the MDR, while TE(3,1,2) and TM(1,3,2) are supported by the mixture of ED, MD, EQ, MQ, EO, and MO. Figure 2c demonstrates that TE(3,1,1) and TM(1,3,1) are governed by ED and MQ, MD and EQ, respectively. From the analysis, the guided mode resonances are the superposition of different electric and magnetic multipoles. Meanwhile, the Mie resonance is mainly supported by one type of multipoles. The superposition of multipoles results in distinct behaviors: some are tightly confined within the structure, constituting bound states, while others can propagate into the far field, functioning as leaky channels. This feature allows resonances sharing the same leaky channel to interact and induce transformations in the multipolar composition, giving rise to phenomena such as an anti-crossing type feature and the emergence of Friedrich-Wintgen BIC^38^. One example is the anti-crossing and crossing regions between TE(3,1,1) and TM(1,3,1) while tuning from Γ point to X point as demonstrated in Section II of Supporting Information. Besides, the quantum number *l, m, n* of each guided-mode resonance represents the node number of each calculated field distribution in x, y, and z dimensions, respectively (shown in Fig. 2d). Meanwhile, the coupling between MDR and TM(1,3,2) can be discovered in the field distributions, where two nodes at the top layer and both sides of the nanodisk disappear in the magnetic field distribution of TM(1,3,2), but emerge in the distribution of MDR. Moreover, the electric and magnetic types of guided-mode resonance are orthogonal to each other, as seen in the field distributions. This property causes different behaviors for electric and magnetic types in the resonance splitting phenomenon.

**Fig. 3:**
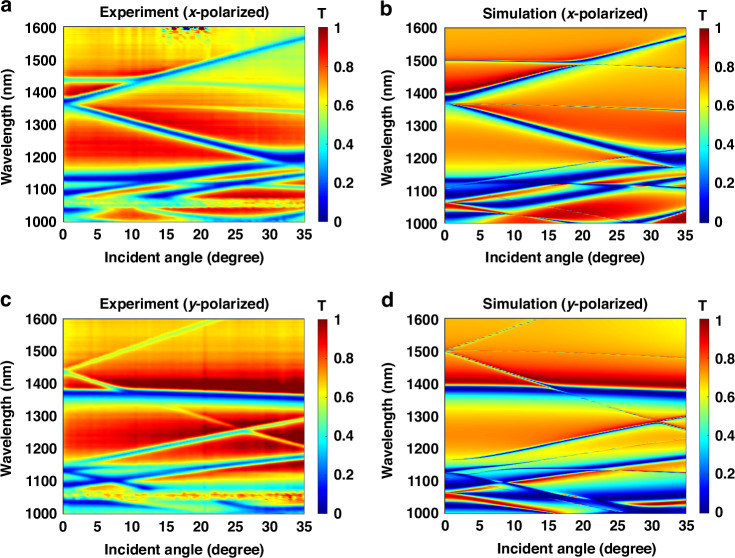
The angular response of guided-mode resonances. The measured (**a**, **c**) and simulated (**b**, **d**) transmission spectra under *x*-polarized (**a**, **b**) and *y*-polarized (**c**, **d**) optical illumination with the incident angle tuned along *y*-axis. The experimental transmission spectra are obtained by multiplying the measured transmission of the metasurface with the substrate reference and the measured transmission of the substrate with the air reference

The calculated band structure (Fig. 1b) shows the mode splitting of M1 and M2, causing two coupling regions between M1 and M2 when moving from the Γ point to the X point (orange circles). In the measured and simulated transmission spectra (Fig. 3), we observe resonance splitting and coupling between TE(3,1,1) and TM(1,3,1) while tuning the incident angle along *y*-axis under *x-* and *y*-polarized illumination. Via tuning the incident angle along the *y*-axis, the TE mode remains and splits under *x* and *y*-polarized incidences, respectively (in Fig. 3). The split resonance TE(3,1,1) or TM(1,3,1) would split into three different resonances in the spectrum: one goes upwards, another downwards, and the third one remains almost constant with decreasing Q factors when varying the incident angle. Meanwhile, the TM mode splits and remains under *x-* and *y*-polarized incidences, respectively. The different behaviors of resonance splitting for the TE and TM resonances originate from the symmetry of enhanced electromagnetic fields. The Si metasurface itself is a symmetric system with ${C}_{z}^{4}$ symmetry, indicating that the whole structure is invariant after rotating along the *z*-axis for 90° ($\varepsilon \left(x,y,z\right)={C}_{z}^{4}\varepsilon (x,{y},{z})$). However, the incident electromagnetic field is less symmetric with ${C}_{z}^{2}$ symmetry, which enables the enhanced electromagnetic fields to be negative after rotating along *z*-axis for 180° (${\bf{E}}\left(x,y,z\right)=-{C}_{z}^{4}{\bf{E}}\left(x,y,z\right)$). The resonant electromagnetic field allows the controllable resonance splitting via tuning the incident polarization and incident angle in the *x*- or *y*-axis. Additionally, the spectral location of TE (3,1,1) exhibits a discrepancy between the measured and simulated transmission spectra, potentially stemming from differences in the silicon refractive indices between simulations and experiments, as well as slight fabrication imperfections such as roughness or size of the fabricated silicon disks of the metasurface.

Here, we add more details to demonstrate the resonance splitting phenomenon. No matter whether the incident illumination is linearly-polarized along the *x*- or *y*-axis, the directions of the phase accumulation for electric and magnetic types are orthogonal. When tuning the incident angle along the *y*-axis of *y*-polarized light illumination, the magnetic field of the incident light remains nearly unchanged. In contrast, the electric field varies with different incident angles, resulting in the split feature of the TE resonance while the TM resonance remains stable with increasing incident angle. Vice versa when the incident beam is *x*-polarized. Since one of TE(3,1,1) and TM(1,3,1) splits while the other remains, the coupling between them always occurs while tuning the incident angle regardless of the polarization of the incident beam. We have demonstrated all the optical properties of the Si metasurface in the linear region, including the band structure, multipolar analysis, transmission spectra, and incident angle dependence. Moreover, due to the high sensitivity of guided-mode resonances to incident angles, the experimental characterization may be susceptible to the spatial coherence of both the illumination and imaging systems. Specifically, the selection of the spot size and divergence of the used light source, in addition with the numerical aperture of optical components, would be limited when measuring k-vector-sensitive resonances. This phenomenon has been observed in several works^39–41^. In our experiments, a supercontinuum broadband laser (SuperK Fianium-15) with around 100 µm width spot onto the metasurfaces has been used for measuring the transmission. More detailed information can be accessed in the Materials and Methods section.

Next, we demonstrate the ability of the designed metasurface for FWM emission and imaging using a homemade nonlinear optical setup, as presented in the Supporting Information (Fig. S6 of Section III). The pump and signal inputs are femtosecond laser beams with 150 fs pulse and 80 MHz repetition rate. The signal beam first passes through the imaging target and then is mixed with the pump beam via a dichroic mirror. The two beams are focused onto the metasurface sample by an aspheric lens with a focal length of 5 cm. The converted image was collected by a 20 × objective with *NA* = 0.4, and was detected by a CCD camera and spectrometer (Ocean Insight, NQ512-2.5) for measuring the emission intensity. The strong light-matter interactions facilitated by guided-mode resonances can significantly enhance the nonlinear generation. In the remaining section, our emphasis lies on utilizing guided-mode resonance TE (3,1,2) at the pump wavelength to enhance FWM processes.

**Fig. 4:**
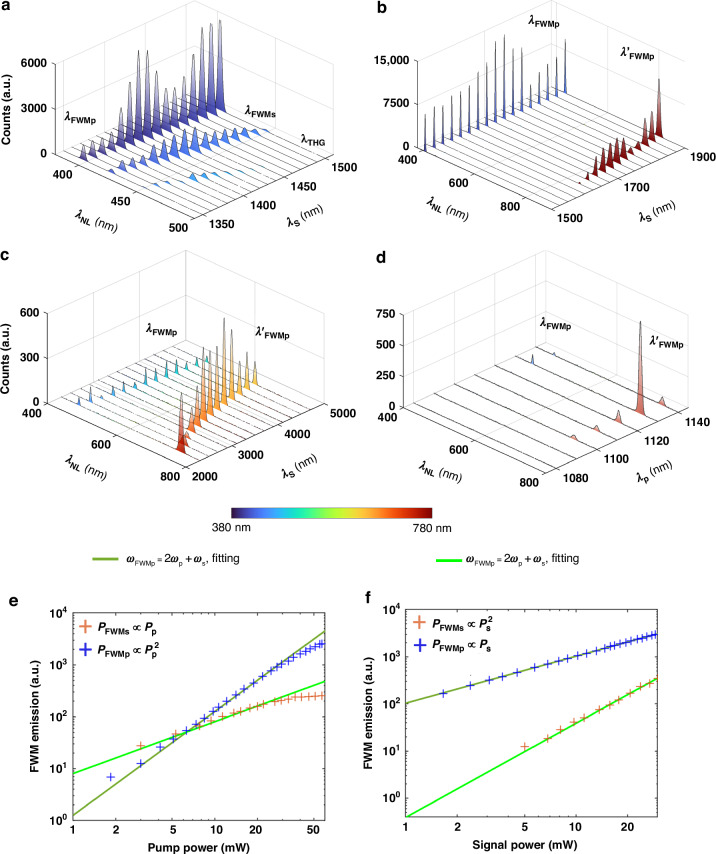
The nonlinear emissions from the Si metasurface and power dependence between input power and nonlinear emissions. **a**–**c** The measured FWM and THG emission spectra from Si metasurface while tuning the signal wavelength (*λ*_s_) when fixing the pump wavelength *λ*_*p*_ = 1130 nm. The tuning ranges of the signal wavelength are (**a**) from 1350 to 1500 nm, (**b**) from 1500 to 1900 nm, and (**c**) from 2300 to 4700 nm. **d** The measured FWM emission spectra from Si metasurface while tuning the pump wavelength (*λ*_p_) from 1080 nm to 1140 nm. The signal beam wavelength is fixed at *λ*_*s*_ = 2250 nm. The relation between the frequencies of FWM emissions, signal frequency, and pump frequency can be expressed as: *ω*_FWMp_ = 2*ω*_pump_ + *ω*_signal_, *ω*_FWMs_ = *ω*_pump_ + 2*ω*_signal_, and *ω’*_FWMp_ = 2*ω*_pump_ − *ω*_signal_. The THG emission is generated by the signal beam (*ω*_THG_ = 3*ω*_signal_). The color indicates the perceived color of human eye for the wavelength range 380 − 780 nm. **e**, **f** The quadratic and linear power dependence of pump beam (**a**) and signal beam (**b**) on the two FWM processes (*ω*_FWMp_ = 2*ω*_pump_ + *ω*_signal_ and *ω*_FWMs_ = *ω*_pump_ + 2*ω*_signal_) with experimental data and fitting curves

We first present the measured nonlinear emission spectra of third-harmonic generation (THG) and FWM induced by the signal beam and pump beam. Nonlinear emissions are measured under normal incidence for the signal and pump beams with different central wavelengths, as shown in Fig. 4a–d. We first fix the pump wavelength at 1130 nm (resonant position of TE(3,1,2)) and tune the signal wavelength from 1350 to 1500 nm in Fig. 4a. Due to the eased phase-matching condition of metasurfaces, we can directly observe two FWM processes and one THG process from signal beam enhanced within the Si metasurface, whose nonlinear emission components with respect to the pump and signal frequencies can be expressed as *ω*_FWMp_ = 2*ω*_pump_ + *ω*_signal_, *ω*_FWMs_ = *ω*_pump_ + 2*ω*_signal_, and *ω*_THG_ = 3*ω*_signal_. Here, we define the FWM process led by the pump beam as *λ*_FWMp_, the FWM process led by the signal beam as *λ*_FWMs_, and the THG emission of the signal beam as *λ*_THG_. When the signal wavelength is at the spectral position of TE(3,1,1) at 1440 nm and TM(1,3,1) at 1375 nm, the FWM emissions and THG emission are strongly enhanced. Here, we fix the input pump power at 43 mW, which is higher than the fixed input signal power of 25 mW, so that the pump beam can further enhance the FWM process (*λ*_FWMp_). As predicted, due to the strong enhancement of pump beam, the nonlinear emission of FWM process *λ*_FWMp_ is much stronger than the FWM process of *λ*_FWMs_, and the emission of *λ*_FWMs_ is stronger than the THG process of *λ*_THG_. Furthermore, we fix the pump wavelength at 1130 nm and tune the signal wavelength from 1500 nm to 1900 nm, which is off the resonant position of TE(3,1,1) and TM(1,3,1). As shown in Fig. 4b, the two FWM processes can be observed in the spectra, marked as *ω*_FWMp_ and *ω’*_FWMp_. The relationships between frequencies can be calculated as *ω*_FWMp_ = 2*ω*_pump_ + *ω*_signal_ and *ω’*_FWMp_ = 2*ω*_pump_ −*ω*_signal_. These two FWM processes are significantly enhanced by the pump beam at the resonant position of TE(3,1,2). Owing to the absence of resonant enhancement at the signal wavelength, the FWM emission of *λ*_FWMs_ and the THG emission of the signal beam *λ*_THG_ are too weak to be observed. For more details, the simulation results of THG and FWM emissions, related nonlinear field distribution, and nonlinear multipolar structure are included in Section IV of Supporting Information. Aiming for broadband nonlinear imaging, we further investigate the FWM emissions under the signal beam in a longer wavelength range as demonstrated in Fig. 4c. The pump beam is fixed at 1130 nm, the resonant position of TE(3,1,2). The central wavelength of the signal beam is tuned from 2250 nm to 4700 nm, covering the NIR to MIR spectral range. Figure 4c exhibits relatively strong FWM emissions among a large range of signal wavelengths, where no high-Q resonance is observed in the transmission spectra (See Fig. S12 in Section V of the Supporting Information). By varying the pump wavelength from 1080 nm to 1140 nm while maintaining the signal wavelength at 2250 nm, we demonstrate that TE(3,1,2) guided resonance significantly amplifies the two FWM processes (*ω*_FWMp_ and *ω’*_FWMp_) without any resonant responses with the metasurface at the signal beam wavelength. As a result, the FWM emissions reach the peak at a pump wavelength of 1130 nm, which is more than tenfold higher than the FWM emissions off the resonance TE(3,1,2) noticed in Fig. 4d. The conversion efficiency of FWM based on the Si metasurface is measured and calculated, included in Section VI of the Supporting Information.

Theoretically, both FWM emissions, as shown in Fig. 4d, have a quadratic relation to the pump input power and a linear relationship to the signal input power. Here, fixing the pump and signal beams at *λ*_*s*_ = 1420 nm (for *λ*_*FWMs*_), *λ*_*s*_ = 1700 nm (for *λ*_*FWMp*_) and *λ*_*p*_ = 1130 nm, we change the power of pump and signal inputs and then measure the emission of two FWM processes *ω*_FWMp_ and *ω*_FWMs_ (Fig. 4e, f). Using a nonlinear least-squares solver to implement the curve-fitting of measured data, we demonstrate the quadratic and linear relations between the FWM emissions and input powers. Based on the pump beam’s quadratic relationship, the pump beam can significantly enhance the FWM emission. Thus, the reliance on high signal power input can be reduced, expanding its application in the nonlinear imaging field. Additionally, when the pump input power is increased to around 30 mW, the pump power dependence in FWM processes reaches the saturation region. This phenomenon has been observed in Si metasurfaces for THG^30,42^.

**Fig. 5:**
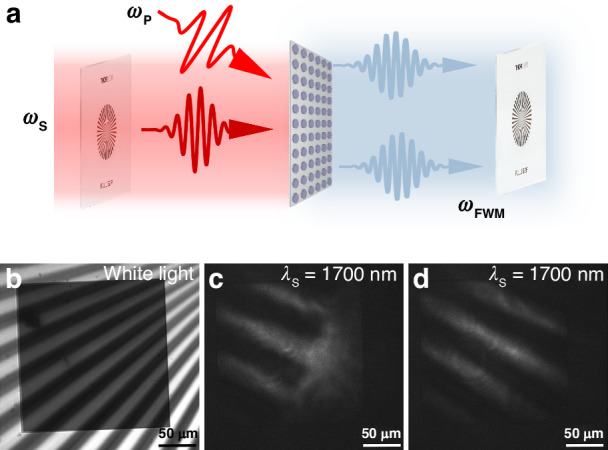
The schematic of the infrared imaging technique based on the Si metasurface and its converted imaging with the signal wavelength at 1700 nm. **a** The schematic diagram of infrared imaging based on FWM via the Si metasurfaces. **b** The image of the target and metasurface under white light source illumination. **c**, **d** Transformed visible images of the target via metasurfaces under signal and pump beam illumination with the signal wavelength. The pump wavelength is fixed at 1130 nm

We then demonstrate the ability to apply our Si metasurface for nonlinear imaging (Fig. 5). We set the pump wavelength at 1130 nm to enhance the FWM process and set the signal wavelength at 1700 nm. Then we use the lens groups to expand both the signal and pump beam, creating a large beam spot (around 200 µm width) covering the whole metasurface. Figure 5b shows the visible images when the Si metasurface overlaps with an NBS 1963A resolution test target under white light illumination. Figure 5c, d gives the converted images captured by a scientific CCD camera (CS165MU/M, Thorlabs) under the illumination of pump and signal beams, in which the pattern has been converted into visible with clear edges based on the FWM processes (*λ*_FWMp_ and *λ*^′^_FWMp_). For details about the specific image areas of the target, see Section VII in the Supporting Information. We have successfully achieved the IR imaging with a clear pattern based on FWM via metasurfaces without any resonance enhancing the signal beam. Via the strong enhancement from the pump beam, which can quadratically increase the FWM emission, we believe the signal wavelength range can be further expanded to a much longer wavelength range. As a result, this would enable broadband nonlinear imaging covering both near- and mid-IR spectra.

**Fig. 6:**
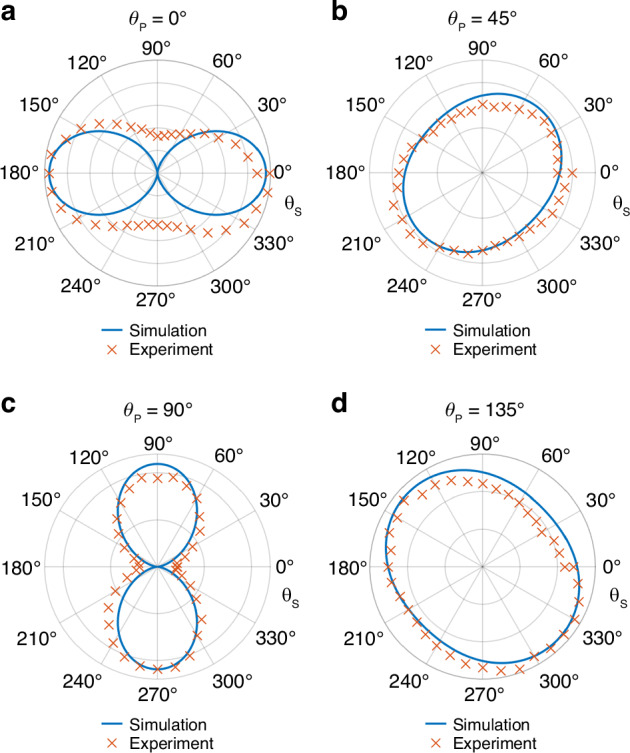
The measured and simulated linear polarization dependence of signal and pump beam on the FWM emission (*ω’*_FWMp_ = 2*ω*_pump_ − *ω*_signal_). The 0º-polarization angle is related to *x*-polarized incidence (**a**). The 45º-polarization angle is related to the incidence polarized 45º with respect to the x axis (**b**). The 90°-polarization angle is related to *y*-polarized incidence (**c**). The 135°-polarization angle is related to the incidence polarized 135º with respect to the x axis (**d**)

The IR light emitted from an IR object carries a wealth of information about the target, including the wavelength, polarization, intensity, phase, and coherence of the scattered light from the target. Thus, the dependence of nonlinear emission on the input polarization is directly linked to the optical polarization properties of the IR target. In the following, we explore the polarization dependence of FWM emissions on the polarization of the pump and signal beams. The polarization direction of the pump and signal beams are defined as *θ*_p_ and *θ*_s_, respectively, which are measured between the polarization direction and the *x*-axis. *θ*_p_ is set at 0°, 45°, 90° and 135°, respectively, and *θ*_s_ is tuned from 0° to 360° as elucidated in Fig. 6a–d. When the pump beam is *x*- or *y*-polarized (*θ*_p_ = 0°, 90°), only one of the electric modes (TE(3,1,2) or TE(1,3,2)) gets excited depending on the polarization direction. The electric mode is orthogonal to the electric field polarized in the other direction, resulting in a very sensitive response of the FWM emission to the polarization of signal beam with double oval shapes shown in Fig. 6a, c. When *θ*_p_ = 45°, 135°, the enhanced electric field of the pump beam is the superposition of TE(3,1,2) and TE(1,3,2), leading to a less sensitive response of the FWM emission to the polarization of signal beam with oval shapes in Fig. 6b, d. The results reveal that the sensitivity of the FWM emission to the polarization direction of the signal beam can be well controlled by changing the polarization of the pump beam. Depending on the actual requirement, we can make the nonlinear imaging technique polarization selective or unselective to the signal polarization. A polarization-sensitive FWM emission is also observed when tuning the pump beam polarization. For more experimental details and theoretical analysis, see Sections VIII and IX in the Supporting Information. The polarization of light is a probe of the surface texture of the material as well as the molecular structures. This polarization-sensitive FWM emission may be beneficial for the future, extending the proposed nonlinear imaging techniques with additional functionalities, including the ability to detect materials sensitive to polarization and enhancing the resolution and sensitivity for surface detection.

**Fig. 7:**
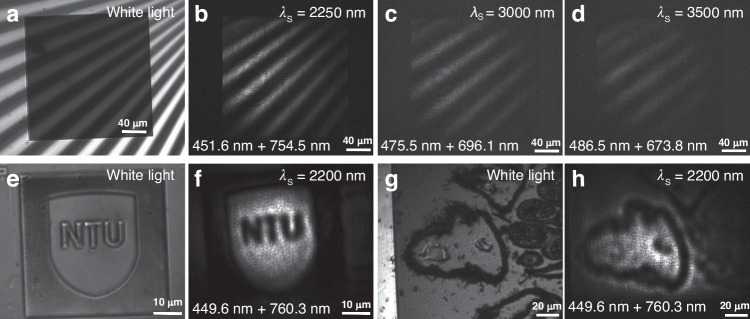
The broadband infrared imaging of various targets. **a** The image of the target and metasurface under white light source illumination. **b**–**d** Transformed visible images of the target via metasurfaces under signal and pump beam illumination with the signal and FWM wavelengths (top: *λ*_s_, bottom: *λ*_FWMp_ + *λ*^′^_FWMp_). The pump wavelength is fixed at 1130 nm. **e** Image of the sample with ’NTU’ marking under white light source illumination. **f** Transformed visible image of the ’NTU’ marking via the Si metasurface under signal and pump beam illumination with the signal and FWM wavelengths. The pump wavelength is fixed at 1130 nm. **g** Image of the biological sample under white light source illumination. **h** Transformed visible image of the ’NTU’ marking via the Si metasurface under signal and pump beam illumination with the signal and FWM wavelengths. The pump wavelength is fixed at 1130 nm

Next, we demonstrate the capabilities of broadband nonlinear imaging based on FWM processes enhanced by the Si metasurfaces. The image when the Si metasurface overlaps with the target under white light illumination is shown in Fig. 7a. Figure 7b–d shows the transformed images captured by the CCD camera under the illumination of the pump and signal beams, in which the pattern has been converted into visible with clear edges based on the FWM processes. More detailed FWM images covering other wavelengths can be seen in Fig. S18 of Section X in the Supporting Information. The pump beam is fixed at 1130 nm to enhance the whole FWM process, and the signal is set from 2250 nm to 4000 nm in Fig. 7c, d and Fig. S18, with the corresponding wavelengths of FWM emissions shown at the bottom of each figure. In this way, we have successfully achieved clear broadband imaging of a pattern in the IR based on FWM via metasurfaces. Notably, the proposed nonlinear imaging technique enables the imaging of arbitrary objects. We further take two examples including a target marked with ‘NTU’ and a sample of biological tissue - pollen of corylus.

Figure 7f, h shows the transformed images under the illumination of the pump and signal beams with a clear pattern of these imaging samples. These results prove that the nonlinear imaging technique based on our Si metasurface can convert the image in the broadband IR range with arbitrary objects into the visible, which enables the direct detection of IR light using visible Si cameras.

The imaging resolution of our infrared imaging technique can reach up to 5 µm, which can be estimated through the bar width in Fig. 7e, f. It’s worth noting that guided-mode resonances, unlike localized modes, can be susceptible to the boundary effect of finite illumination areas^43,44^. This effect can be eased by reducing unit sizes. We believe that the imaging resolution can be further improved by utilizing metasurfaces with more compact lattice sizes and optical components that exhibit reduced chromatic aberrations. Furthermore, the current imaging resolution (∼5 µm) adequately covers a 10 × 10 unit array, where the boundary effect is deemed insignificant to impact the conversion efficiency.

**Fig. 8:**
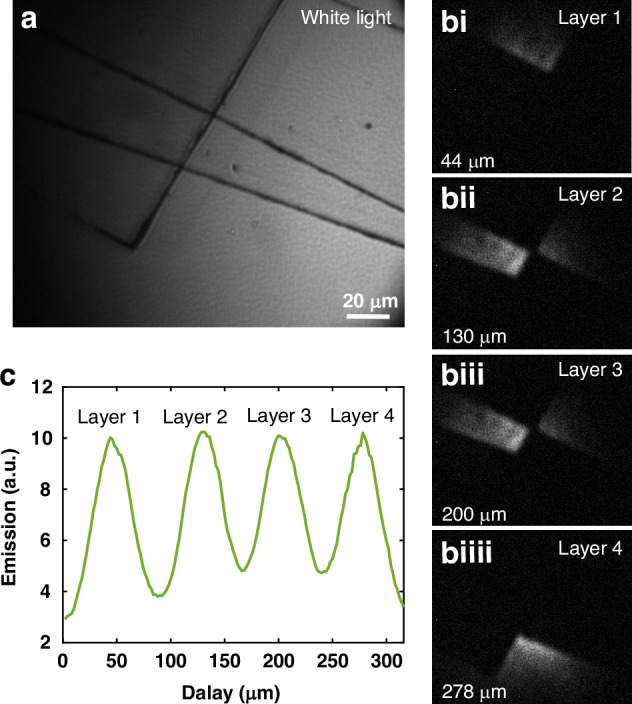
The thickness detection based on the proposed infrared imaging technique via FWM. **a** The images of several glass chips (BK7) overlapped together under white light source illumination. **bi**–**biiii** The transformed images show that the nonlinear emissions occur on different area of different layers depending on the moving distance of the delay line (shown below). **c** The normalized density of the FWM emission with the length of the time delay

Furthermore, our infrared imaging technique supported by two pulsed lasers shows sensitivity to object thickness, which allows for the detection of object thickness based on the overlapping of signal and pump pulses in the spatial domain. By changing the delay line of the optical setup, the time delay of the pump beam can be accurately controlled. Meanwhile, the imaging object would supply a time delay to the signal beam depending on the imaging sample’s thickness and refractive index. Here, we measure the time delay induced by the optical delay line to detect the different thicknesses of the imaging target. We change the time delay per 1 µm. The resolution of the thickness detection is determined by the delay line’s minimum incremental movement and the laser’s pulse duration. We first overlap several thin glass chips as shown in Fig. 8a to arrange several imaging areas with different layers. Then, via illuminating the signal and pump beam, the FWM emissions occur at different areas while tuning the time delay, as illustrated in Fig. 8bi–biiii. We normalize the intensity of the FWM emission with the length of the time delay so that the distance between each layer of glass chips can be measured, as shown in Fig. 8c. As a result, we can detect that the distance between each layer is around 175.5 µm, 142.9 *µ*m, and 159.2 µm, considering the refractive index of glass and air is 1.49 and 1.0 at the working laser wavelength, respectively. We believe this technique can be further improved by increasing the signal-to-noise ratio of the detector, using a laser with a narrower bandwidth, and increasing the resolution of the delay line. This method introduces a novel way to examine the internal surfaces of objects, including paintings and artworks, without direct contact. Moreover, choosing a signal beam wavelength that can penetrate the medium also facilitates non-invasive sensing and imaging of targets through opaque materials.

## Discussion

The infrared imaging technique via nonlinear III-V semiconductor metasurfaces has been recently investigated^11,45^. However, the III-V semiconductors suffer from challenging fabrication approaches^46^. Alongside this, they are limited to second-order nonlinear interactions such as SHG and sum frequency generation (SFG). Here, we propose a new platform for realizing broadband infrared imaging via silicon metasurfaces through FWM. Silicon metasurfaces are widely investigated with nanofabrication techniques routinely available in optics and electronics communities (for CMOS). Moreover, the quadratic relationship between the pump input power and nonlinear emissions in FWM processes allows for more efficient nonlinear generation. The latter is particularly important for nonlinear imaging via metasurfaces with naturally low conversion efficiencies by means of compensation from the pump intensity. Remarkably, the pump wavelength in FWM can be further away (e.g., in SWIR), which reduces the noise level in the visible range, where the output image takes place. We first design a Si metasurface with an array of nanodisks on top of a thin Si film. This metasurface can support multiple guided-mode resonances and one Mie resonance in the near-infrared range. Multiple resonance splittings have been observed and demonstrated both theoretically and experimentally. The FWM and THG emissions of signal and pump beams are measured and calculated, including tuning the signal and pump wavelengths, powers, polarizations, and time delays. We observe strong FWM enhancement across a broadband signal beam wavelength range governed by the guided-mode resonance at the pump wavelength. Consequently, we achieve broadband IR imaging of arbitrary objects using a visible Si camera. As a result, by designing the high-Q guided-mode resonance located at the pump wavelength, the employment of FWM can further reduce the dependence on high-power signal input in nonlinear imaging. We expect that our proposed FWM imaging technique will produce considerable breakthroughs in realizing next-generation all-optical infrared imaging and spectroscopy devices.

## Materials and methods

### Numerical simulations

The field patterns, transmittance spectra, and multipolar analysis are calculated using the finite element method (FEM) in COMSOL Multiphysics 6.2 software. The band structure calculation is based on the Massachusetts Institute of Technology PhotonicBands (MPB) open sources^47^. The model, including a Si nanodisk on top of a Si film with a SiO_2_ substrate beneath, is formulated under periodic boundary conditions for all simulations. Field distributions within the Si nanodisks and the Si film serve as inputs for calculating the multipolar analysis. To analyze the multipolar structure in the presence of a substrate in our case, we use the polarization currents induced inside the Si nanostructures and consider the multipoles of the metasurface via spherical multipole decomposition to calculate the contributions associated with each multipole^37^. For simplicity, the refractive index of SiO_2_ is set as 1.5 in our simulations, and the refractive index of Si is set as 3.4 for wavelength above 1000 nm. For wavelengths under 1000 nm, due to the absorption and variation of silicon dispersions, we used the experimentally measured data of dispersions in our simulations.

### Metasurface fabrication

Amorphous silicon metasurface with a thickness of 500 nm (including Si film and nanodisks) was fabricated on a quartz substrate with a thickness of 1 mm. First, plasma-enhanced chemical vapor deposition deposited a 500 nm thick amorphous silicon layer on the quartz substrate. Subsequently, a resist layer (ZEP520) is spin-coated on the wafer. Then, the periodic patterns were defined in the resist layer with electron beam-lithography. Using the resist as a mask layer, the patterns were transferred to the silicon diaphragm by the inductively coupled plasma technique. Finally, the remaining resist was removed with the Nmethyl-2-pyrrolidone (NMP) liquor.

### Linear transmission measurement

The transmission spectra of the fabricated metasurface sample were measured using a home-built broadband laser (SuperK Fianium-15) spectroscopy setup in a confocal configuration. A supercontinuum broadband laser (SuperK Fianium-15) was used as the light source. The light was focused by an aspheric lens with a focal length of 7.5 cm to a beam waist of around 100 *µ*m on the sample. By using a Mitutoyo M Plan NIR 10x NA = 0.26 objective, the light transmitted through the sample was then collected and directed to the spectrometer (Ocean Insight, NQ512-2.5). Specification for NQ512-2.5 Ocean Insight spectrometer: Wavelength range: 900 nm to 2.45 µm, Optical resolution: 6.48 nm FWHM.

### Nonlinear optical experiments

A custom-built FWM imaging setup is detailed in the Supporting Information (Fig. S6 of Section III). Pump and signal inputs consisted of femtosecond laser beams with 150 fs pulse duration and an 80 MHz repetition rate. A delay line comprising two prisms allowed precise control over the pump beam’s time delay by adjusting the distance between them. Both pump and signal beams were overlapped using a beam splitter and focused onto the metasurface sample via an aspheric lens with a focal length of 5 cm. The resulting image was captured by a 20 × objective with NA = 0.4 and detected using a CCD camera (CS165MU/M, Thorlabs) and spectrometer (Ocean Insight, QEPRO-XR) for measuring emission intensity. When achieving FWM images, two types of lens with focal lengths of 10 cm and 3.5 cm are used for composing the lens groups, enlarging the laser spot of the signal and the pump beam to ~100 *µ*m on the metasurface. The lens groups were removed during the measurement of the FWM conversion efficiency. The signal and pump beams are focused onto the metasurface sample by a 10 × objective with NA = 0.26 with a spot size of around 26 µm.

Specification for CS165MU/M CCD camera: Monochrome CMOS, 1440 × 1080 Pixel (1.6 MP) Sensor with 3.45 µm Square Pixels, imaging area: 4.968 mm × 3.726 mm, pixel size: 3.45 *µ*m x 3.45 *µ*m. Peak Quantum Efficiency: 69% at 575 nm. Specification for QE Pro Ocean Insight spectrometer: Wavelength range: 200 nm to 950 nm, Optical resolution: 1.6 nm FWHM.

## Supplementary information

Supporting Information: Broadband Infrared Imaging Governed by Guided-Mode Resonance in Dielectric Metasurfaces

## Data Availability

The data presented in this study are available from the following source: 10.17631/10.17631/RD-2024-0010-DDAT.
