# Supplementary material for: Broadband infrared imaging governed by guided-mode resonance in dielectric metasurfaces
Source: Light Sci Appl. 2024 Sep 10;13:249. doi: 10.1038/s41377-024-01535-w (PMC11387824; doi:10.1038/s41377-024-01535-w)
Supplement: Supplementary file 1 — Supporting Information: Broadband Infrared Imaging Governed by Guided-Mode Resonance in Dielectric Metasurfaces [file 41377_2024_1535_MOESM1_ESM.docx]

**Supporting Information:**

Broadband Infrared Imaging Governed by Guided-Mode Resonance in Dielectric Metasurfaces

Ze Zheng^1^, Daria Smirnova^2^, Gabriel Sanderson^1^, Cuifeng Ying^1^, Demosthenes C. Koutsogeorgis^3^, Lujun Huang^4^, Zixi Liu^5^, Rupert Oulton^6^, Arman Yousefi^1^, Andrey E. Miroshnichenko^7^, Dragomir N. Neshev^2^, Mary O’Neill^3^, Mohsen Rahmani^1*^, Lei Xu^1*^

^1^Advanced Optics and Photonics Laboratory, Department of Engineering, School of Science Technology, Nottingham Trent University, Nottingham, NG11 8NS, UK.

^2^ARC Centre of Excellence for Transformative Meta-Optical Systems (TMOS), Research School of Physics, The Australian National University, Canberra, ACT 2601, Australia.

^3^School of Science Technology, Nottingham Trent University, Nottingham, NG11 8NS, UK.

^4^Extreme Optoelectromechanics Laboratory (XXL), East China Normal University, Shanghai, 200062, China.

^5^School of Physics, Nankai University, Tianjin, 300071, China.

^6^Department of Physics, Imperial College London, London, SW7 2BW, UK.

^7^School of Engineering IT, University of New South Wales, Canberra, ACT 2600, Australia.

*Corresponding author(s). E-mail(s): mohsen.rahmani@ntu.ac.uk; lei.xu@ntu.ac.uk.

**OUTLINE:**

1. Band structure
2. Multipolar structure of eigenmodes
3. Experimental Setup for nonlinear measurements
4. Simulation of THG/FWM with Si disk-film metasurface
5. Simulated transmission spectrum of Si metasurface from 2000 to 5000 nm
6. Conversion efficiency
7. Polarization dependence on the FWM emission
8. The field distributions of the pump with different polarized incidences
9. The transformed images from the infrared spectral range based on FWM
10. The transformed images from the infrared spectral range based on FWM
11. **Band structure**

We first calculate the band structure for a Si film by introducing an artificial but virtual periodic boundary condition with pitch size $p_{x}=p_{y}=a$. Here, we consider the 1^st^ and 2^nd^ order modes for quantum number $n$. We employ the guided mode expansion for calculating the dispersion relation of the eigen modes via Legume [Inverse Design of Photonic Crystals through Automatic Differentiation, ACS Photonics 2020, 7, 7, 1729–1741]. Figure S1 gives the calculated band structure for a Si film with thickness 500 nm. In general, the guided modes supported by the Si film can be designed at any spectral region by choosing the artificial period, providing a robust platform for the nanoscale light-matter interactions. These modes folded back to the $\Gamma$ point due to the artificial periodicity, can be treated as the ideal bound states in the continuum, To access these modes from external freespace light illumination, additional perturbation is needed to open leaky channels and transform these guided modes into leaky modes with the Q-factors can be tuned based on the strength of the perturbation.


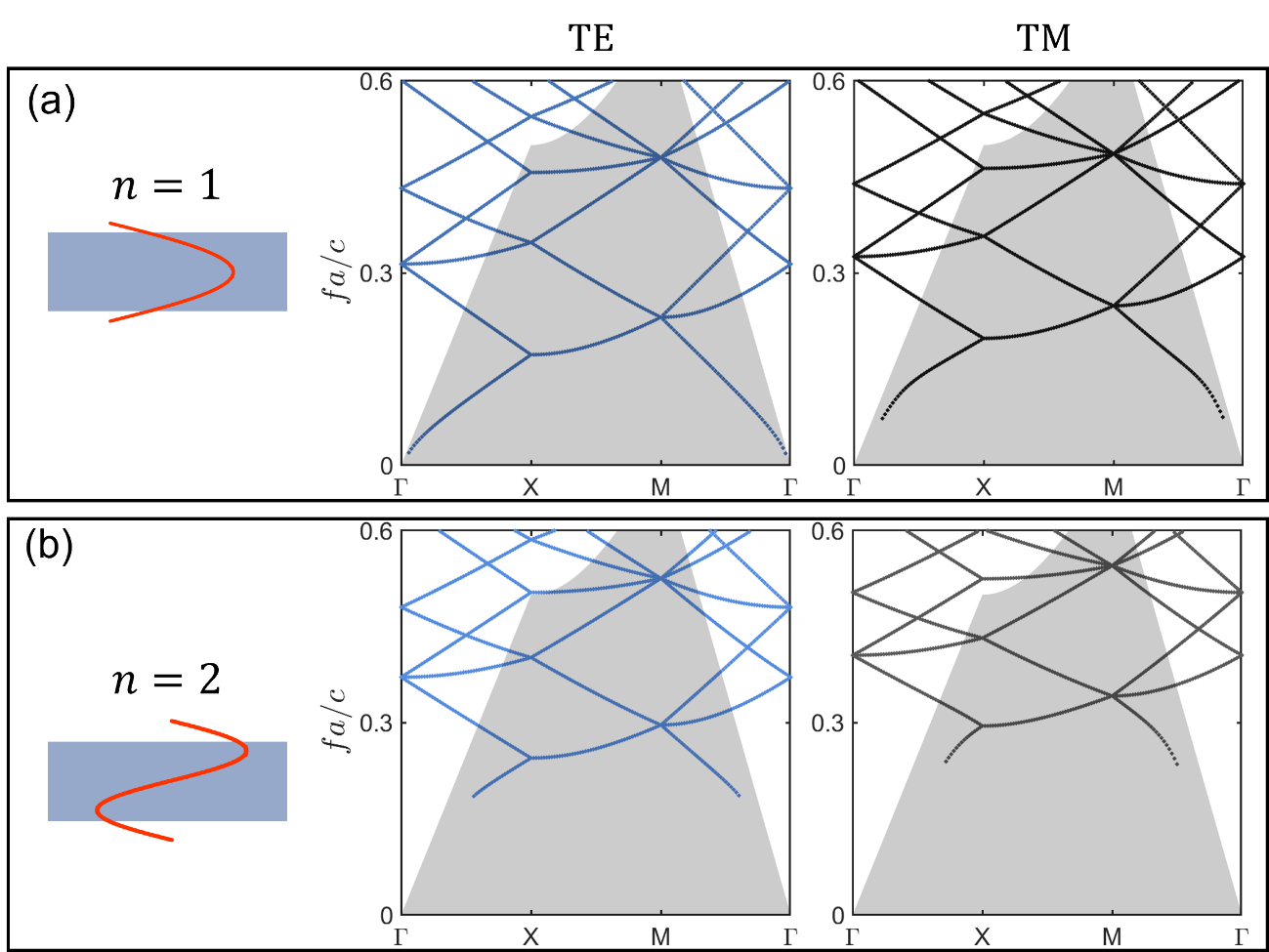


Figure S1. Calculated Band structure for a Si film for TE/TM modes with $n=1$ (a) and TM/TM modes with $n=2$ (b). The gray shading region indicates the area located below the free-space light cone.

Here, as an example, we introduce the Si disk-film metasurface which consists of Si disk array on top of a Si film. Such a system allows accessing a rich variety of different electric- and magnetic-type of eigen modes in specified spectral region. As a comparison, Figure S2 gives the calculated band structure for a Si film, Si disk metasurface and Si disk-film metasurface for the region from $\Gamma$ to X. As can be seen, for our wanted pump spectral region (above 1000 nm), a Si disk metasurface with the same thickness and the same unit cell size only supports less than 4 modes that can be externally excited by plane wave incidence (Figure S2b), while for the Si disk-film metasurface, there are various transformed leaky modes can be accessed under plane wave incidence (Figure S2c), this also matches well with the experimentally measured transmission spectra (Figure 3 in the main text). Importantly, owing to the perturbation for the silicon structure along *z* direction, modes with different quantum numbers $n$ are de-orthogonalised, and enable the possibility to engineer the multiple content and radiation feature of these modes through their coupling.


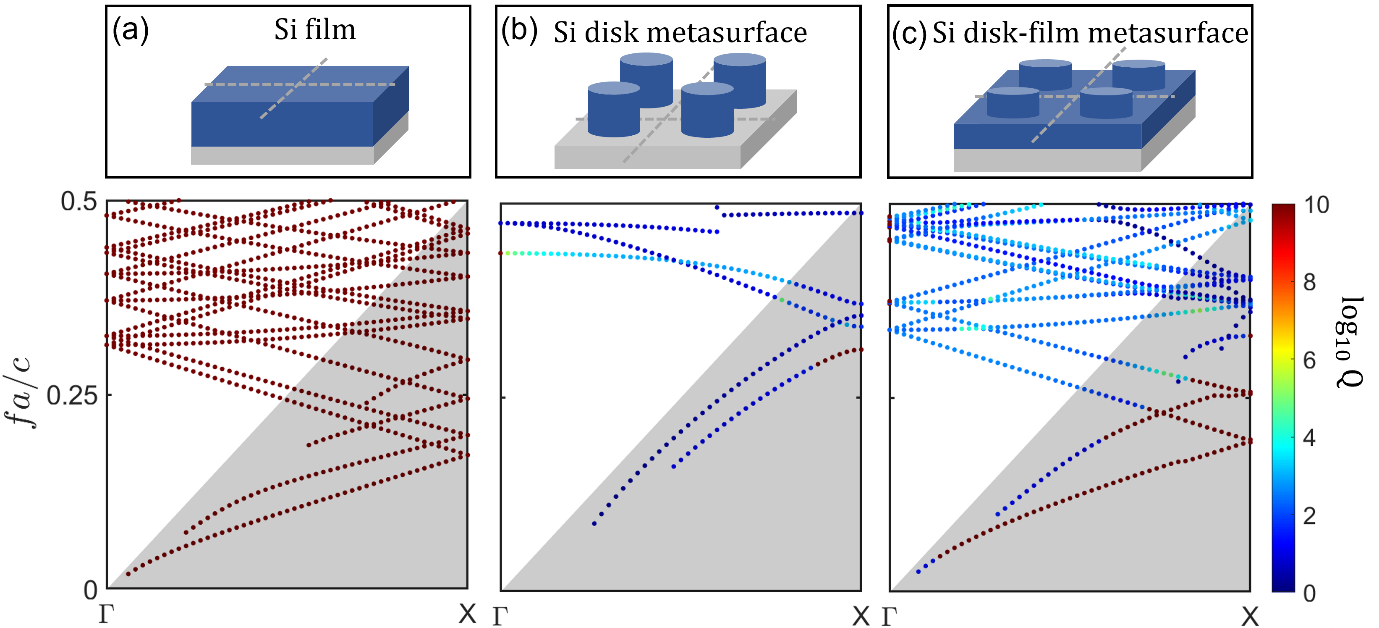


Figure S2. Calculated Band structure for a Si film (a), Si nanodisk metasurface (b) and the proposed Si array-film metasurface (c). The grey dashed line in (a) indicates the introduced virtual periodicity with the same unit size as in (b) and (c).

1. **Multipolar structure of eigen modes**

The optical properties and interference feature can be elucidated through a multipolar approach, where each mode is conceptualised as a superposition of different electric and magnetic multipoles. The periodic structure of the metasurface results in distinct behaviors of these multipoles: some are tightly confined within the structure, constituting bound states, while others can propagate into the far-field, functioning as leaky channels. Notably, modes sharing the same leaky channel can interact and induce transformations in the multipolar composition, giving rise to phenomena such as anti-crossing, exception points type of feature. As an example, we calculated the multipolar content across several bands within specified spectral range, as depicted in Figure S3 – S5.


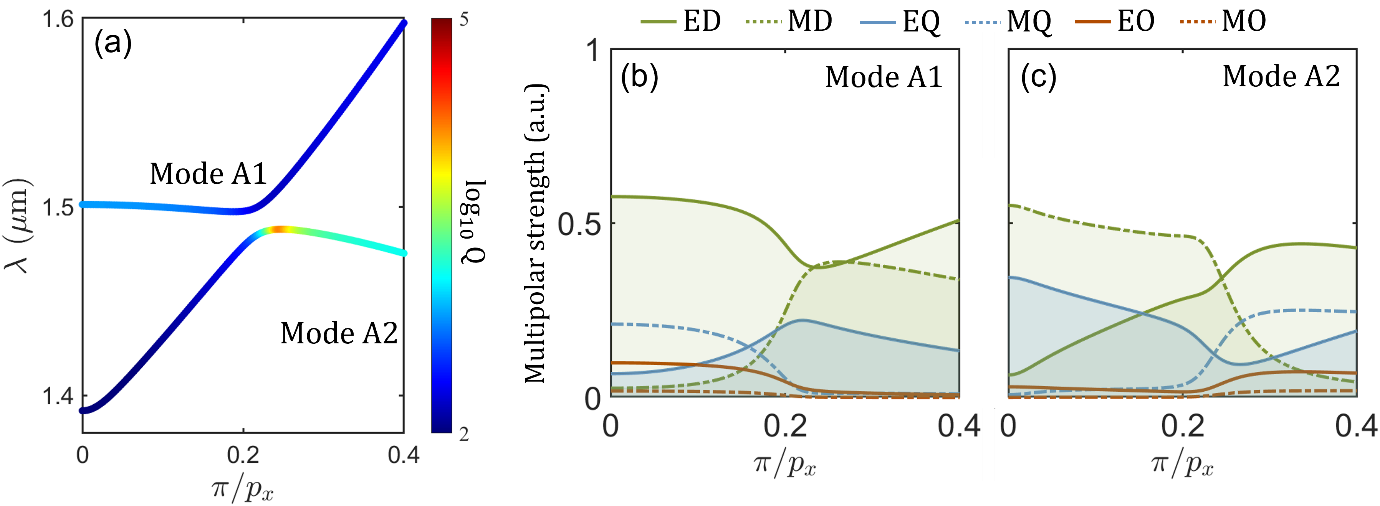


Figure S3. (a) Dispersion of mode A1 and A2. (b) Multipolar content evolution for mode A1 with increasing transverse wavevector $k_{x}$. (c) Multipolar content evolution for mode A2 with increasing transverse wavevector $k_{x}$.


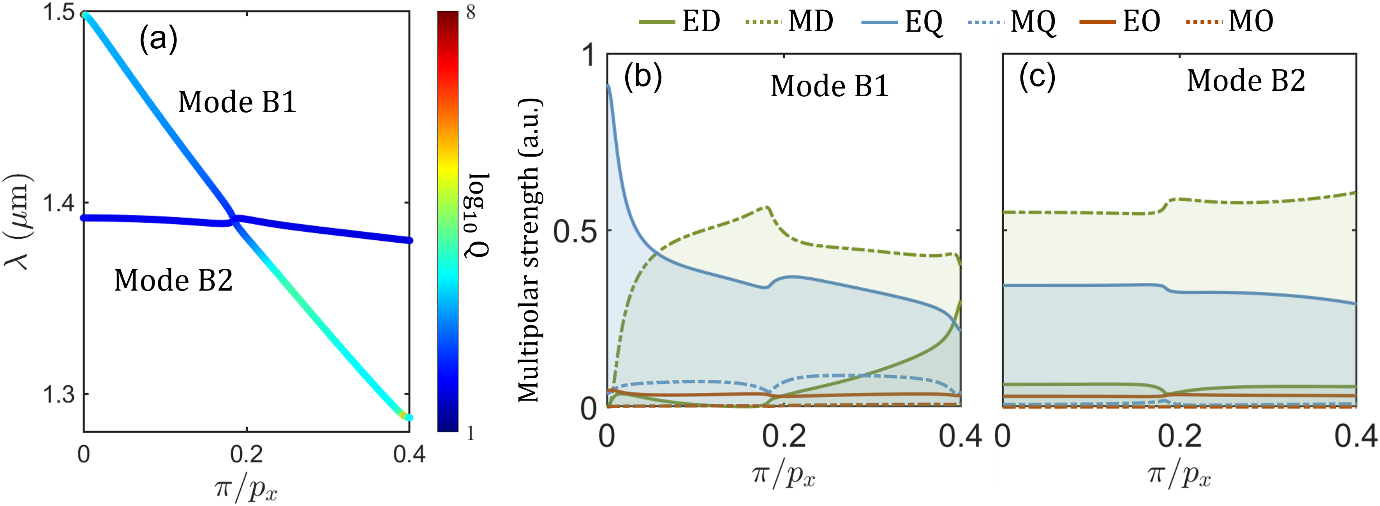


Figure S4. (a) Dispersion of mode B1 and B2. (b) Multipolar content evolution for mode B1 with increasing transverse wavevector $k_{x}$. (c) Multipolar content evolution for mode B2 with increasing transverse wavevector $k_{x}$.


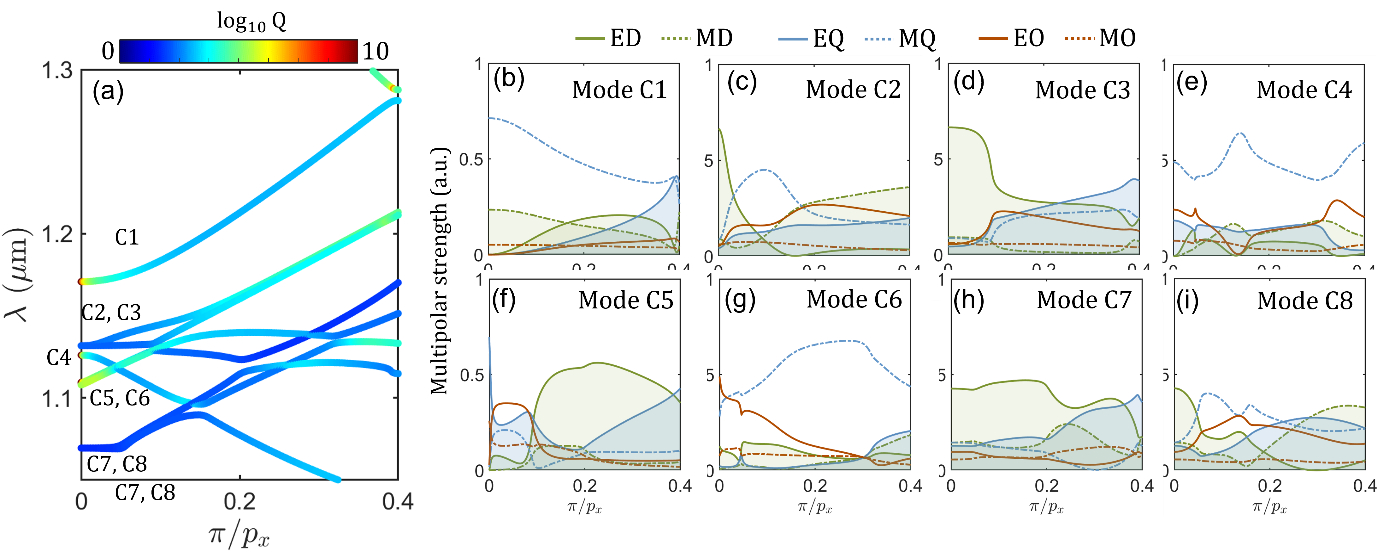


Figure S5. (a) Dispersion of modes C1 – C8. (b - i) Multipolar content evolution for mode C1 – C8 with increasing transverse wavevector $k_{x}$.

1. **Experimental Setup for nonlinear measurements**

The schematic of the experimental setup for measuring the nonlinear signals and nonlinear imaging characterization with the silicon mesurfaces is shown in Figure S6.

**
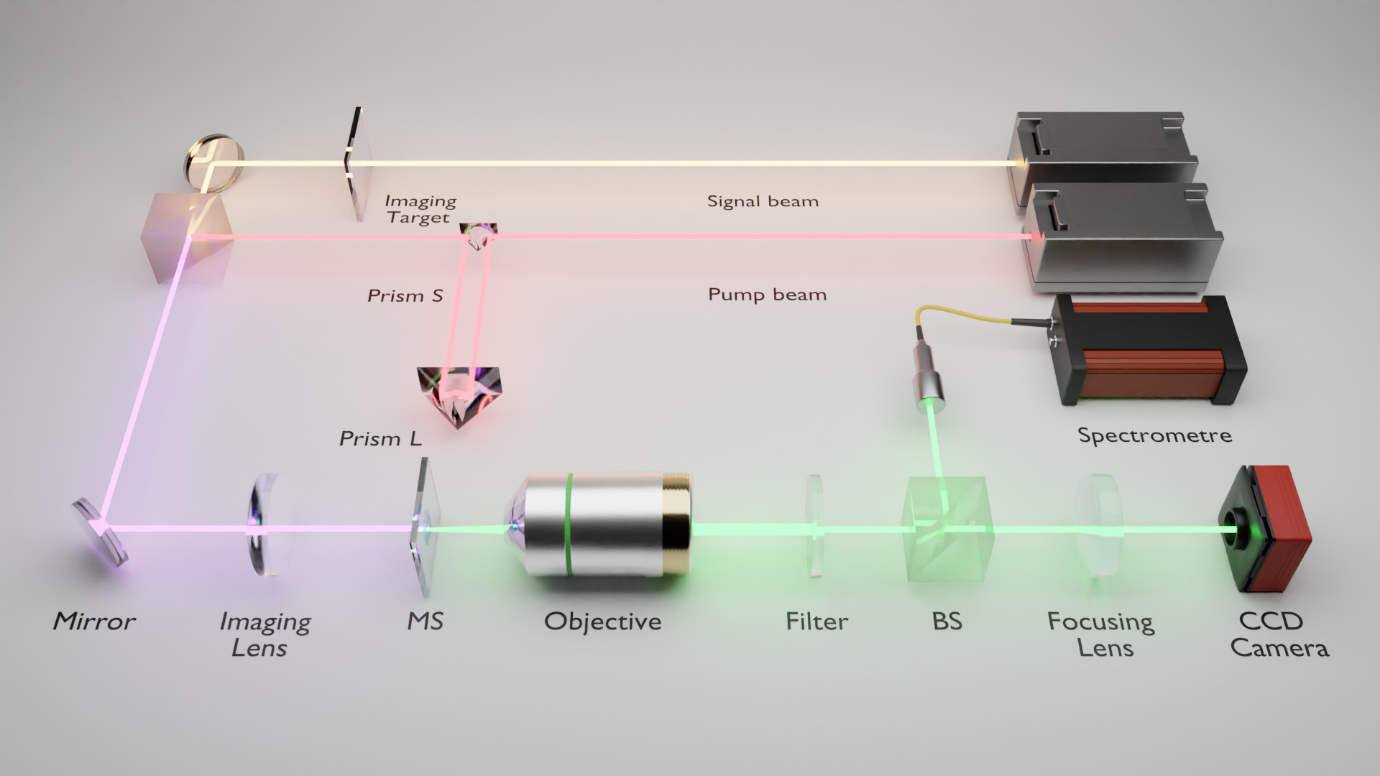
**

Figure S6. The schematic diagram of the experimental setup for nonlinear imaging. The figure is created based on Blender 3.1.0 using an optical components pack provided by Ryo Mizuta Graphics.

1. **Simulation of THG/FWM with Si disk-film metasurface**

The nonlinear responses of our nanodisks were modeled numerically using the finite-element method in COMSOL Multiphysics in the frequency domain. We assumed the undepleted pump field approximation and followed two steps to model the nonlinear response [Optics express 23 (20), 26544-26550 (2015); ACS Photonics 3, no. 8 (2016): 1468-1476]. The electric field at the pump and signal beam were simulated first, and the nonlinear polarization induced inside the Si disk-film metasurface was obtained. We then employed the obtained nonlinear polarization as a source for the electromagnetic simulation at the harmonic wavelength to obtain the generated THG/FWM field. For our amorphous silicon material, the nonlinear susceptibility tensor $\chi^{(3)}$ was considered as a constant scalar value with $\chi^{(3)}=2.45\times{10}^{-19} m^{2}V^{-2}$ [Phys. Rev. B 41, 1542–1560 (1990); Opt. Express 14, 5524–5534 (2006)]. The induced nonlinear polarization components for THG can be simplified as

$\boldsymbol{P}^{\mathrm{THG}}=\varepsilon_{0}\chi^{\left( 3 \right)}\boldsymbol{E}(\boldsymbol{E\cdot E})$,

and the induced nonlinear polarization components for FWM process $\omega_{\mathrm{FWM}}=2\omega_{p}-\omega_{s}$ can be simplified as

$\boldsymbol{P}^{\mathrm{FWM}}=2\varepsilon_{0}\chi^{\left( 3 \right)}\left( \boldsymbol{E}\left( \omega_{p} \right)\cdot{\boldsymbol{E}\left( \omega_{s} \right)}^{*} \right)\boldsymbol{E}\left( \omega_{p} \right)+\varepsilon_{0}\chi^{\left( 3 \right)}\left( \boldsymbol{E}\left( \omega_{p} \right)\cdot\boldsymbol{E}\left( \omega_{p} \right) \right){\boldsymbol{E}\left( \omega_{s} \right)}^{*}$,

where $\varepsilon_{0}$ is the vacuum permittivity, $\boldsymbol{E}$ is the electric field.

We define the power-independent conversion efficiency as

$\eta_{\mathrm{THG}}=P_{\mathrm{THG}}/P_{\mathrm{FW}}^{3}$ for THG process, and $\eta_{\mathrm{FWM}}=P_{\mathrm{FWM}}/\left( P_{s}P_{p}^{2} \right)$ for FWM process. Here, $P_{\mathrm{THG}}$ / $P_{\mathrm{FWM}}$ is the total generated nonlinear emission power for THG/FWM. $P_{\mathrm{FW}}$ is the input power for the fundamental wave during the THG process, and $P_{s}$, $P_{p}$ are the total input power from the fundamental signal and pump beam, respectively.

Figure S7 gives the calculated THG emission efficiency $\eta_{\mathrm{THG}}$ with the wavelength of fundamental wave gradually increases from 1350 nm to 1550 nm. As can be seen, the THG can be significantly boosted when the fundamental wave is in the vicinity of modes TM(1,3,1) and TE(3,1,1). Notably, for both modes, the THG emission shows a stronger emission in the backward direction. We observe a resonance-suppression of the zero-order forward emission (normal emission) when pumping at mode TE(3,1,1). This may be interpreted by the nature of the resonances at the pump and the harmonics, and the nonlinear interference effects. [Optica 3, no. 11 (2016): 1241-1255; Advanced Science 6, no. 15 (2019): 1802119.].

Figure S8 gives the calculated FWM emission efficiency $\eta_{\mathrm{FWM}}$ with different pump and signal beam wavelengths. We observe a strong FWM enhancement when pumping at mode TE(3,1,2) as compared to the case when pumping at mode TM(1,3,2). This is due to the induced stronger electric near-field enhancement for mode TE(3,1,2), as can be seen from the calculated electric field distributions in Figure S9 and S10. Interestingly, when fixing the pump beam at $\lambda_{p}=1132 \mathrm{nm}$ and scan the wavelength of the signal beam from 1350 nm to 1450 nm, we observe a complete switch of the FWM emission from forward to backward directions. This, in general, can be interpreted based on the nonlinearly generated multipoles and their interference effects [Optica 3 (11), 1241-1255 (2016); ACS nano 14 (2), 1379-1389 (2019); Nano Letters 19 (6), 3905-3911 (2019)].


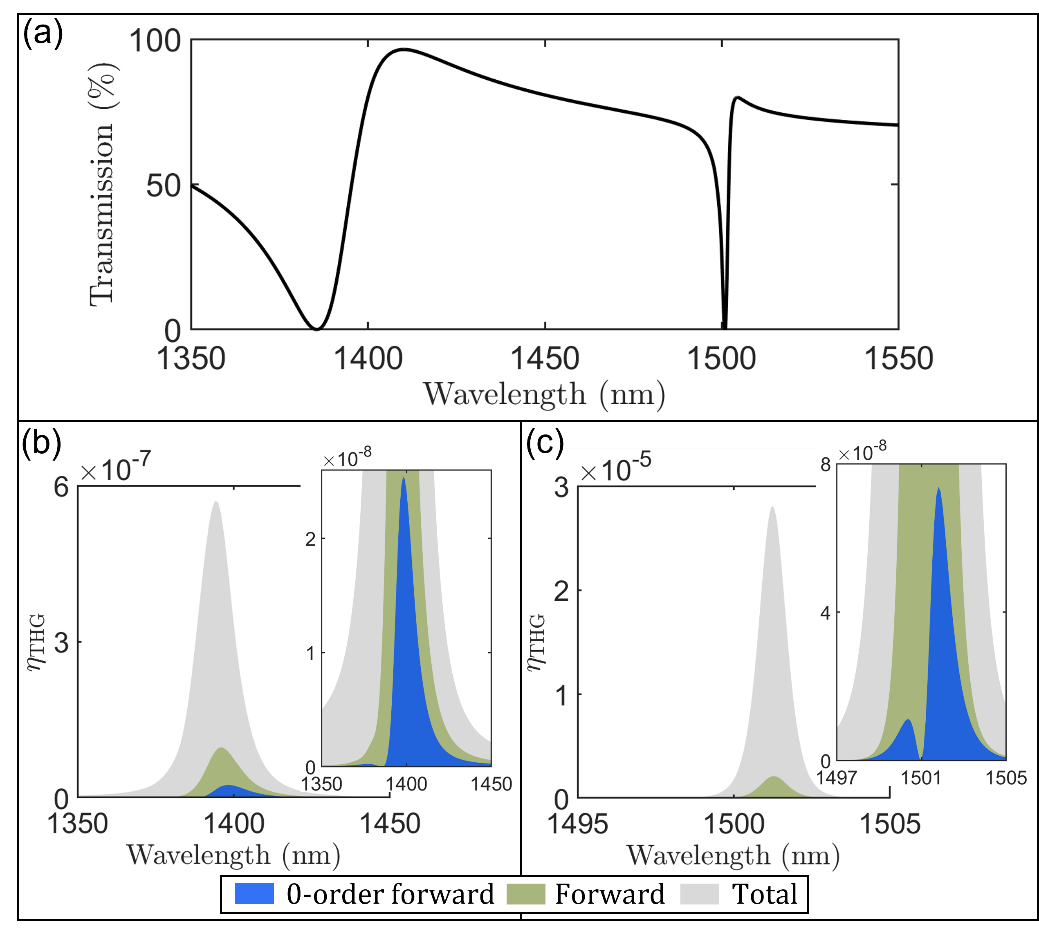


Figure S7. (a) Calculated linear transmission spectrum for the Si disk-film metasurface. (b) Calculated THG efficiency $\eta_{\mathrm{THG}}$ in the vicinity of mode TM(1,3,1). (c) Calculated THG efficiency $\eta_{\mathrm{THG}}$ in the vicinity of mode TE(3,1,1). (ci) gives the enlarged view of the THG emission.


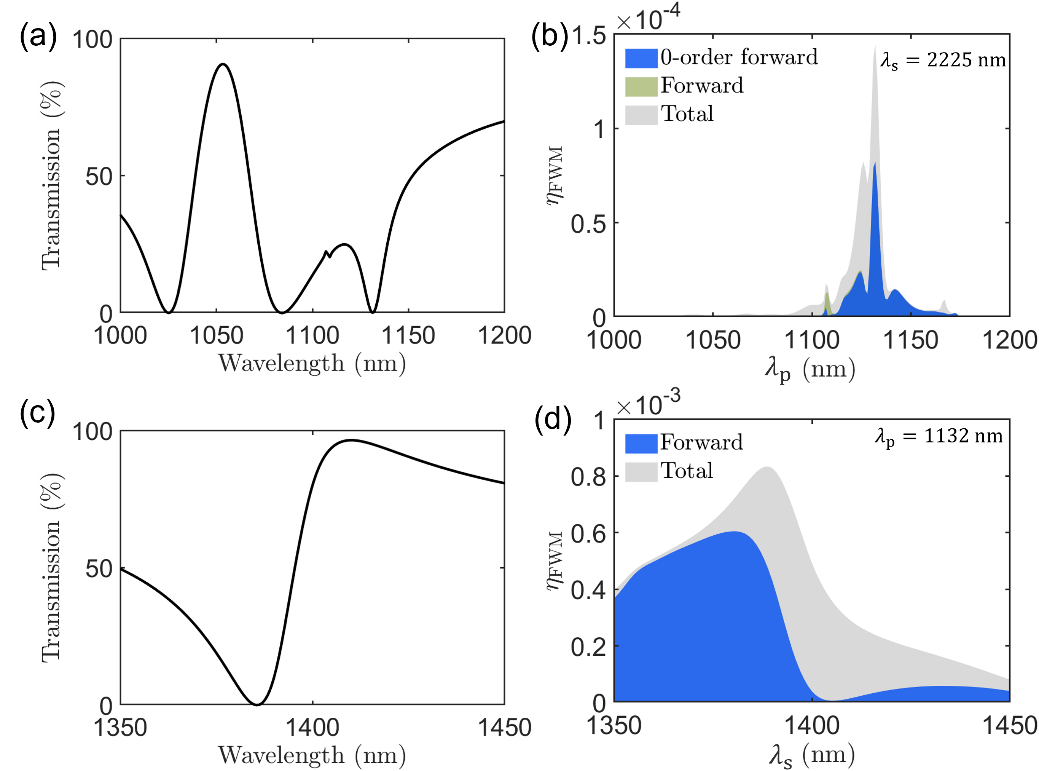


Figure S8. (a) and (c) give the calculated linear transmission spectrum for the Si disk-film metasurface near modes TM(1,3,2), MD, TE(3,1,2) and TM(1,3,1). (b) and (d) give the calculated FWM efficiency $\eta_{\mathrm{FWM}}$ in the vicinity of these modes.


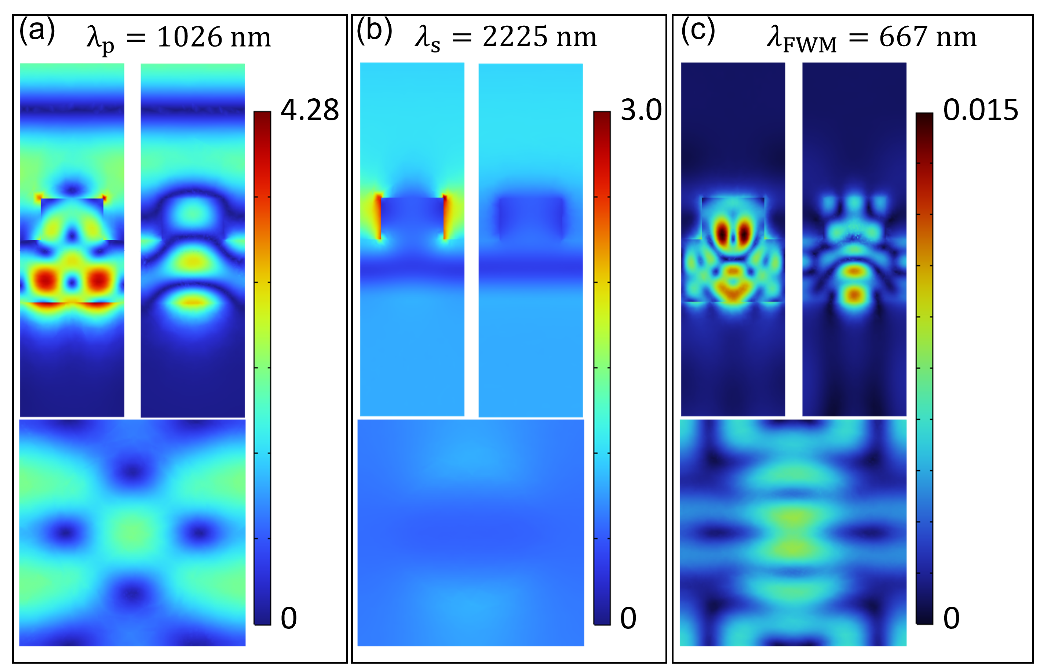


Figure S9. The amplitude of electric near-field distributions $\left| E \right|/E_{0}$ with the Si disk-film metasurface for pump beam $\lambda_{p}=1026 \mathrm{nm}$ (a), $\lambda_{s}=2225 \mathrm{nm}$ (b), and generated FWM field $\lambda_{\mathrm{FWM}}=667 \mathrm{nm}$ (c).


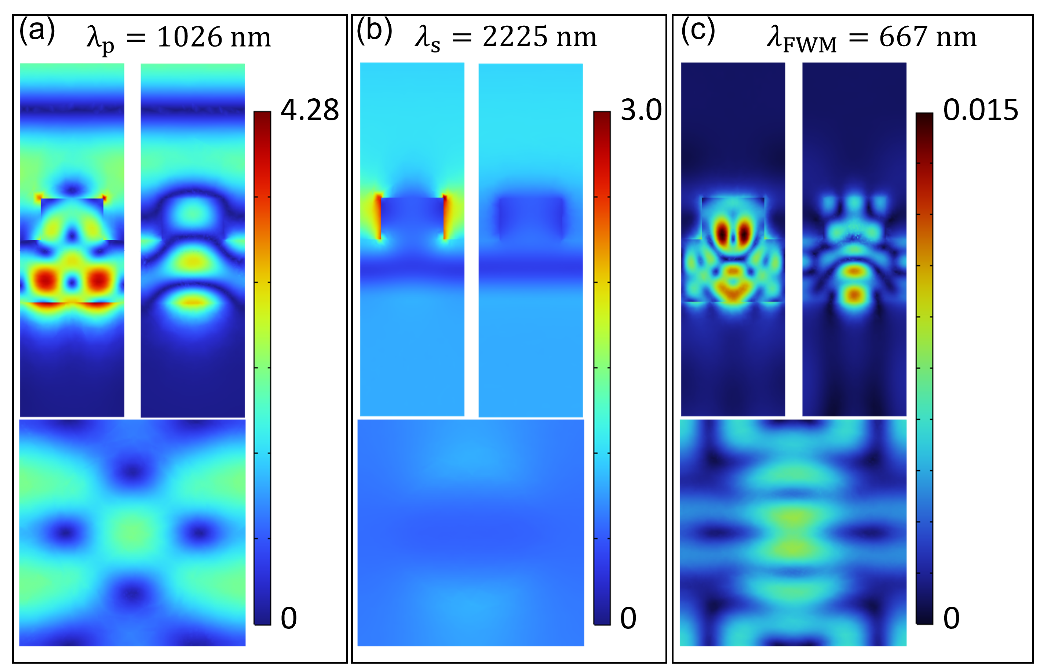


Figure S10. (a, b) The amplitude of electric near-field distributions $\left| E \right|/E_{0}$ with the Si disk-film metasurface for pump beam in the vicinity of mode TE(3,1,2) ( $\lambda_{p}=1132 \mathrm{nm}$ (a)) and generated FWM field $\lambda_{\mathrm{FWM}}=759 \mathrm{nm}$ (b)when the signal beam is in the IR region $\lambda_{s}=2225 \mathrm{nm}$. (c, d) The amplitude of electric near-field distributions $\left| E \right|/E_{0}$ with the Si disk-film metasurface for signal beam in the vicinity of mode TM(1,3,1) ($\lambda_{s}=1405 \mathrm{nm}$ (a)) and generated FWM field $\lambda_{\mathrm{FWM}}=948 \mathrm{nm}$ (b)when the pump beam is in the vicinity of mode TE(3,1,2) ($\lambda_{p}=1132 \mathrm{nm}$).

Figure S11 gives the calculated nonlinear multipolar structure for the generated THG emission and FWM emission, respectively.


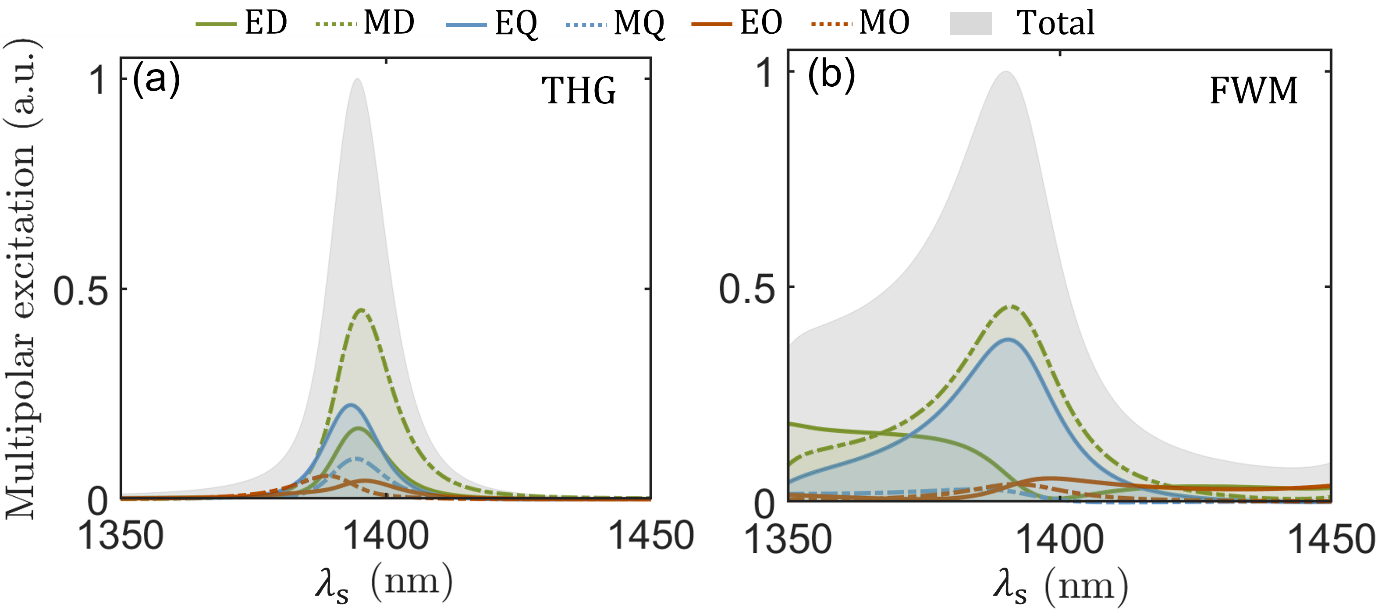


Figure S11. (a) Nonlinear multipolar structure for the generated THG emission when varying the fundamental wave $\lambda_{\mathrm{FW}}$ from 1350 nm to 1450 nm. (b) Nonlinear multipolar structure for the generated FWM emission when varying the signal beam $\lambda_{s}$ from 1350 nm to 1450 nm. Here, the wavelength of pump beam is fixed at $\lambda_{p}=1132 \mathrm{nm}$.

1. **Simulated transmission spectrum of Si metasurface from 2000 to 5000 nm**

The simulated transmission spectrum of Si metasurface from 1500 nm to 5000nm, which indicates no high-Q resonance besides TE(3,1,1) in the spectrum in Figure S12.


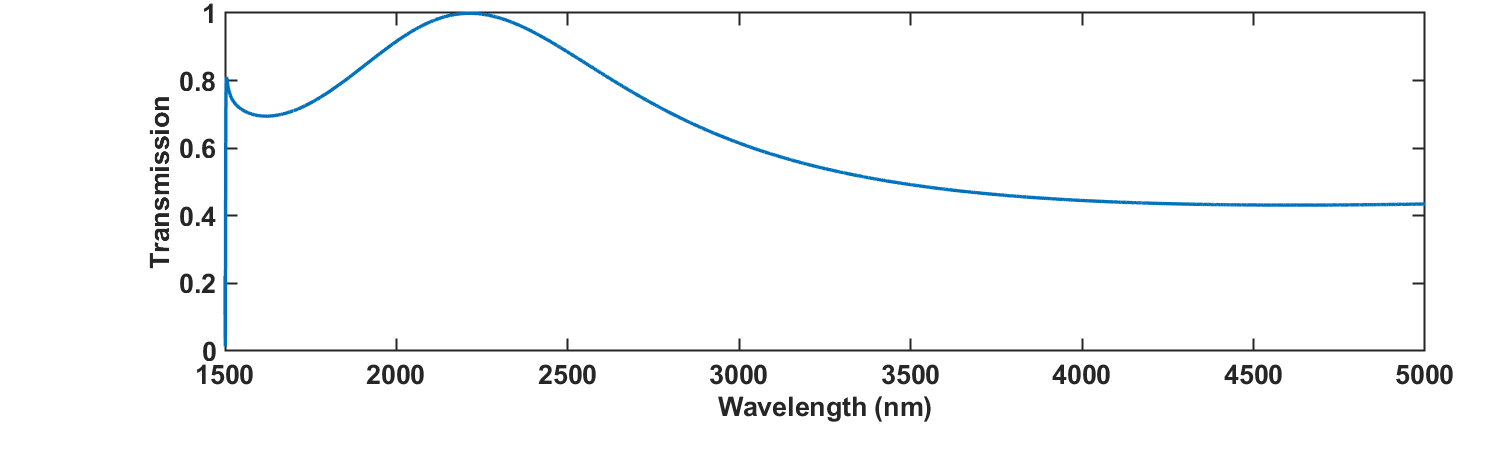


Figure S12. The simulated transmission spectrum of Si metasurface from 1500 nm to 5000nm, which indicates no high-Q resonance besides TE(3,1,1) in the spectrum.

1. **Conversion efficiency**

Next, we demonstrate the measurement of the conversion efficiency with the Si metasurface. The signal and pump beams with central wavelengths of 2250 nm and 1130 nm are focused onto the metasurface sample by a 10× objective with NA = 0.26 with a spot size of around 26 µm. The signal and pump power are 10 mW and 23 mW, respectively. As a result, we have observed the maximum forward FWM emission (0.6nW) via tuning the delay line shown in Figure S13(b). The conversion efficiencies with different difinations can be calculated based on the equations: $\eta_{\mathrm{FWM}}= P_{\mathrm{FWM}}/P_{\mathrm{signal}}P_{\mathrm{pump}}^{2}$ and ${\eta'}_{\mathrm{FWM}}= P_{\mathrm{FWM}}/P_{\mathrm{signal}}$, where $P_{\mathrm{FWM}}$, $P_{\mathrm{signal}}$, and $P_{\mathrm{pump}}$ are respectively the measured power of FWM emission, signal beam, and pump beam. We obtained the conversion efficiencies $\eta_{\mathrm{FWM}}=$ 1.1342 × 10^-4^ W^-2^ and ${\eta'}_{\mathrm{FWM}}=$ 6 × 10^-8^. Moreover, the THG emission is also measured under the illumination of signal beam with input power of 60 mW (Figure S13(a)). Importantly, owing to the strong resonance at the pump, the FWM emission (enhanced via the pump beam) is 80 times higher than the THG emission, without the consideration of the input power difference.


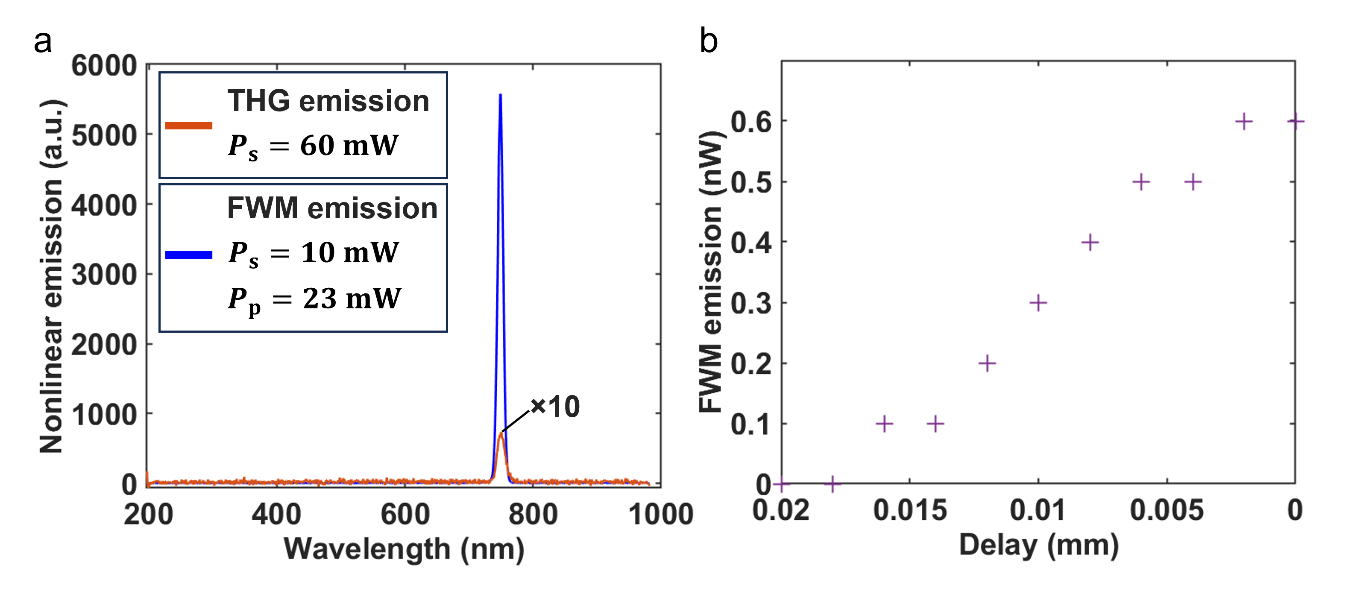


Figure S13. (a) The measured spectra of FWM and THG emissions (${\omega'}_{\mathrm{FWMp}}=2\omega_{\mathrm{pump}}-\omega_{\mathrm{signal}}$; $\omega_{\mathrm{THG}}=3\omega_{\mathrm{signal}}$) under the signal beam with the centre wavelength of 2250 nm and the pump beam with centre wavelength of 1130 nm. The signal and pump powers are 10 and 23 mW when measuring the FWM emission, respectively. The signal power is 60 mW when measuring the THG emission. The THG emission has been multipled for 10 times. (b)The power of FWM emission with tuning the delay between pump and signal pulses.

1. **Polarization dependence on the FWM emission**

Next, we explore the linear polarization dependence of pump and signal on the FWM emissions. The angle is defined as the angle between the x-axis and linear polarization direction. Polarization of the pump is set at 0-, 45-, 90- and 135- degrees respectively, and the polarization of the signal is tuned from 0 to 360 degrees as illuminated in Figure S12. Figure S12(a)(c) demonstrates that the FWM emission have a double-oval shape while tuning the signal polarization angle with the pump beam polarized at 0- and 90- degree. Figure S12(b)(d) indicates that the FWM emission obtains an oval shape while tuning the signal polarization angle with pump polarization of 45- and 135- degree.


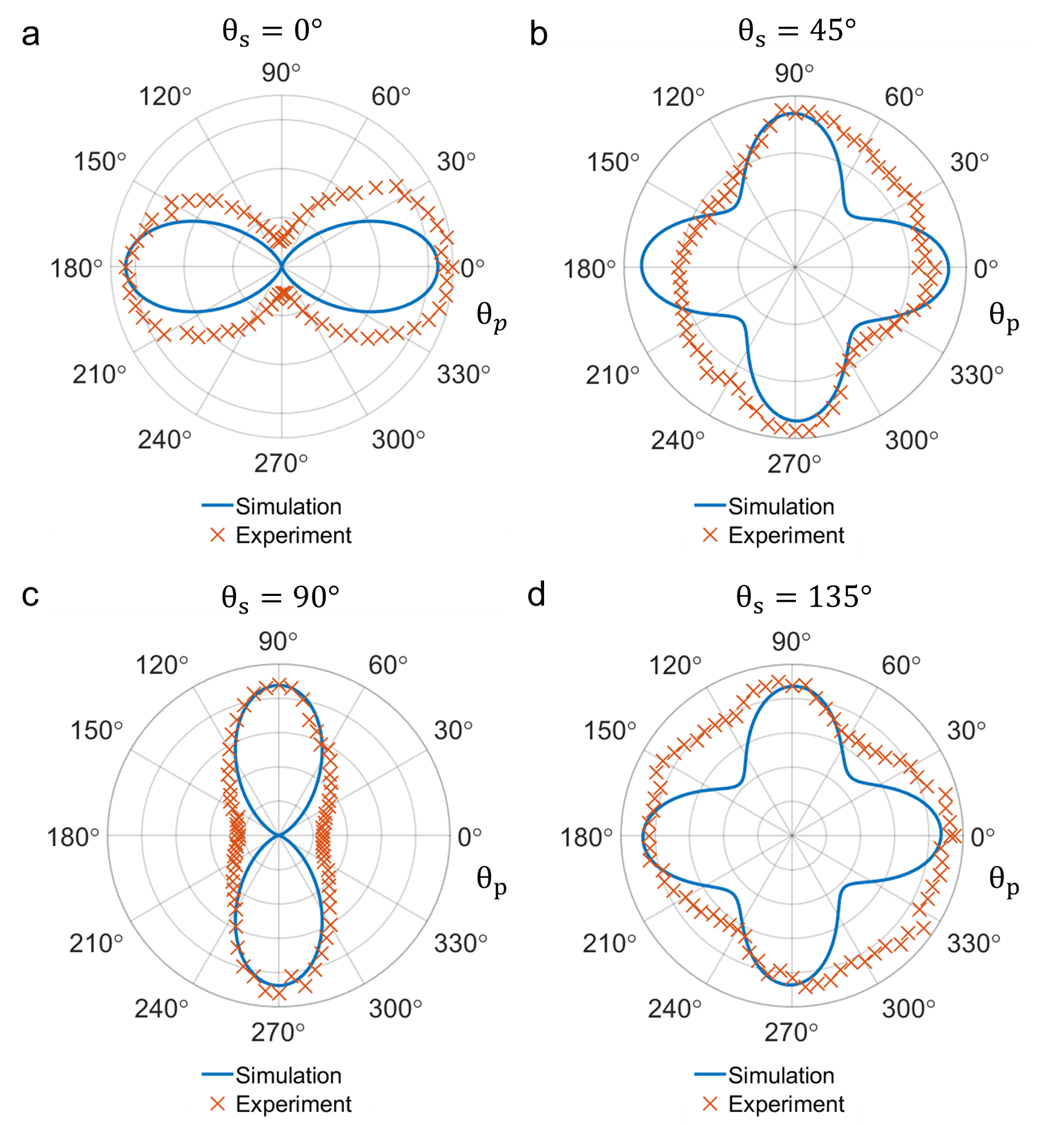


Figure S14. The measured and simulated linear polarization dependence of signal and pump beam on the FWM emission (${\omega'}_{\mathrm{FWMp}}=2\omega_{\mathrm{pump}}-\omega_{\mathrm{signal}}$). The 0-polarization angle is related to x-polarized incidence. The 90-polarization angle is related to y-polarized incidence.

Moreover, a minor discrepancy is evident between 𝜃 = 0° and 𝜃 = 360° in both Figure 6 and Figure S14. This discrepancy arises from the use of half-wave plates to control the linear polarization angle. As the linear polarization is tuned from 0 to 360 degree, the half-wave plate rotates from 0 to 180 degree, leading to slight variations in the beam profile. More details about the power variation from the signal beam can be seen in Figure S15.


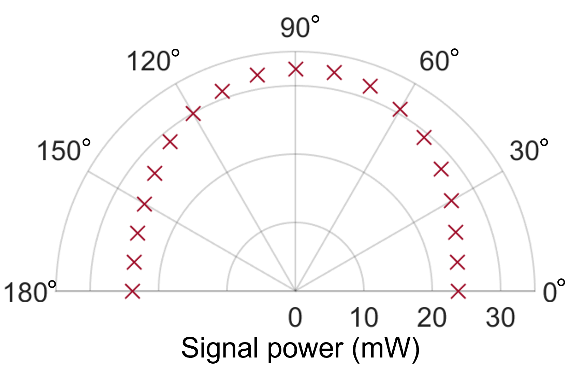


Figure S15. The measured power variation of signal beam after the half-wave plate, tuning with the rotation angle of the half-wave plate.

1. **The field distributions of the pump with different polarized incidences**

Under different polarizations of the incident beam at 1132 nm, two orthogonal guided modes, TE (3,1,2) and TE (1,3,2), are excited. The field distributions for $\theta_{p}=90^{\circ}$ (y-polarized) and $\theta_{p}=45^{\circ}$ (xy-polarized) are depicted in Figure S16. When only one mode is excited ($\theta_{p}=0^{\circ}, 90^{\circ}$), the electric field is predominantly enhanced in one direction, resulting in weak overlapping and FWM emissions with the signal beam polarized in the orthogonal direction. This feature results FWM emissions with high sensitivity to the polarization of the signal beam (as illustrated in Figure 6(a)(c)). Conversely, when both modes are excited ($\theta_{p}=45^{\circ}, 135^{\circ}$), the electric field is enhanced along both x and y directions, leading to low sensitivity of the FWM emissions to the polarization of the signal beam (as revealed in Figure 5(b)(d)).


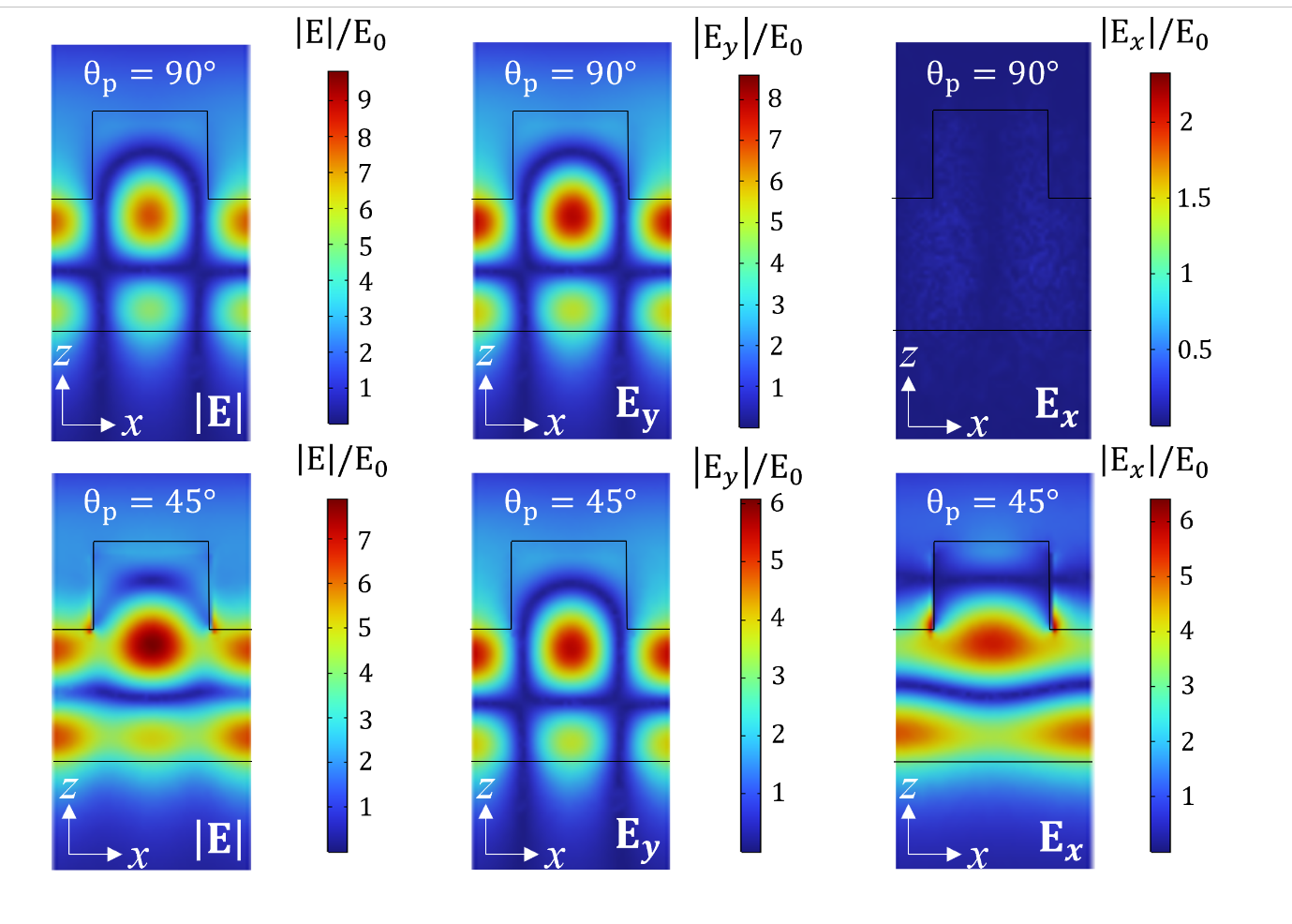


Figure S16. The field distributions when $\theta_{p}=90^{\circ}$ (y-polarized, top) and $\theta_{p}=45^{\circ}$ (xy-polarized, bottom). The left, middle, and right colume represent the total electric field density, the electric field in y direction, and the electric field in x direction.

1. **The imaging target**

The Positive Sector Star Test Target, 72 Bars (R1L1S3P) from Thorlabs has been imaged in Figure 5. Figure S16 demonstrates the specific image areas of each picture in Figure 5(b-d).


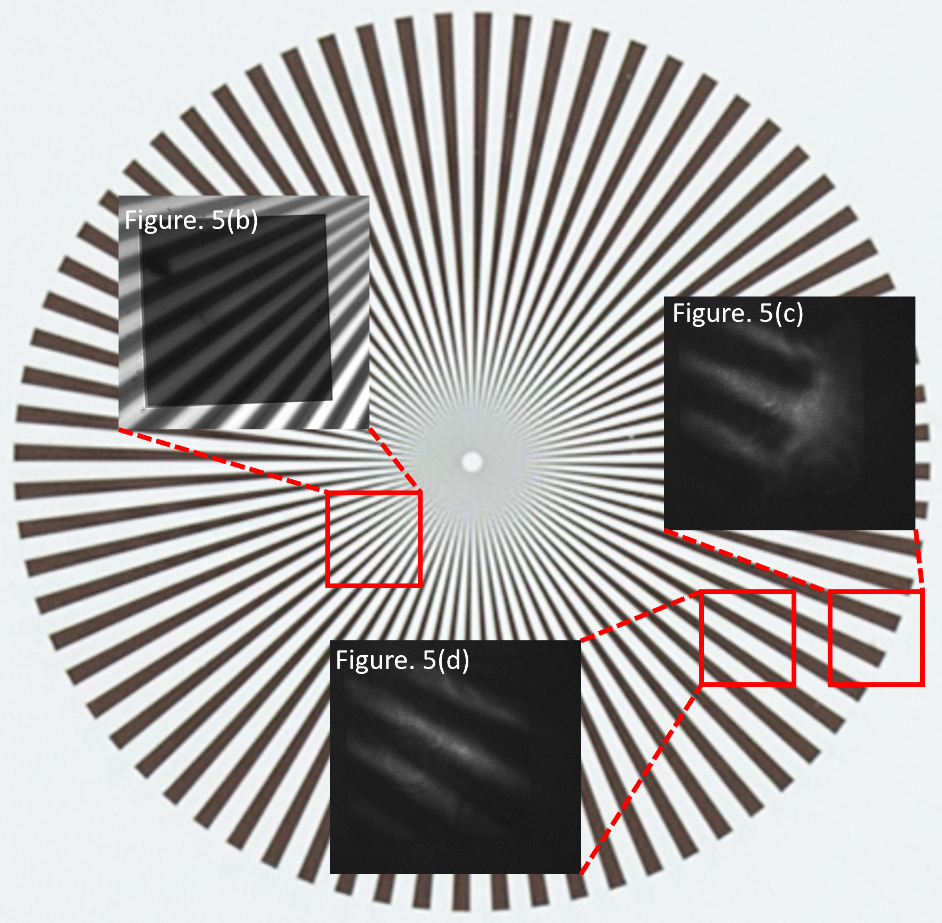


Figure S17. The picture of Positive Sector Star Test Target, 72 Bars (R1L1S3P) from Thorlabs with specific imaging areas (red squares) in Figure 5(b-d).

1. **The transformed images from the infrared spectral range based on FWM**

The transformed images of star imaging target with the illumination of pump beam at 1130 nm and siganl beam from 2250 to 4000 nm in Figure S18.


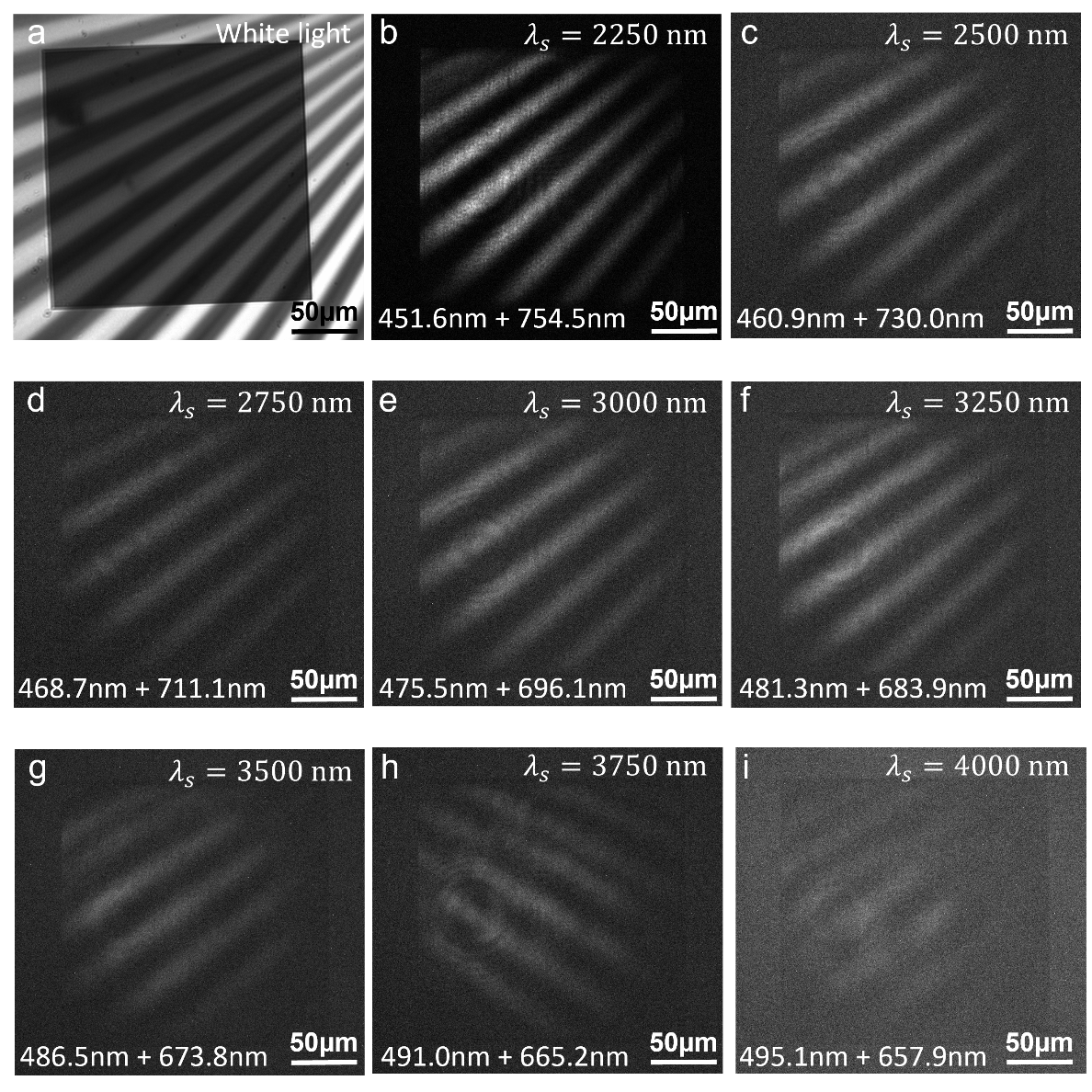


Figure S18. (a) The image of the target and metasurface under white light source illumination. (b-i) Transformed visible images of the target via metasurfaces under signal and pump beam illumination with the signal and FWM wavelengths (top: $\lambda_{s}$, bottom: $\lambda_{\mathrm{FWM}}+{\lambda'}_{\mathrm{FWM}}$). The pump wavelength is fixed at 1130 nm.
